# Risk factors for recurrence and bleeding in colorectal cancer patients with cancer-associated venous thrombembolism

**DOI:** 10.3389/fonc.2025.1648003

**Published:** 2025-08-27

**Authors:** Zhikun Liang, Jieling Mao, Jingwen Xie, Xiaoyan Li, Li Qin

**Affiliations:** ^1^ Department of Pharmacy, the Sixth Affiliated Hospital, Sun Yat-Sen University, Guangzhou, China; ^2^ Biomedical Innovation Center, The Sixth Affiliated Hospital, Sun Yat-Sen University, Guangzhou, China; ^3^ School of Pharmaceutical Science, Sun Yat-Sen University, Guangzhou, China

**Keywords:** colorectal cancer, cancer-associated venous thromboembolism, recurrent thrombosis, bleeding events, patient survival

## Abstract

**Background:**

Colorectal cancer (CRC) patients with cancer-associated venous thromboembolism (VTE) face high risks of recurrence and anticoagulant-related bleeding.

**Objectives:**

Our aim was to assess risk factors associated with recurrence and bleeding and analyze the impact of these outcomes on survival during one-year follow up.

**Design:**

Retrospective study.

**Methods:**

This analysis included consecutive VTE patients treated with anticoagulants from January 2019 to January 2023. The incidence of recurrent VTE, major bleeding (MB), and clinically relevant non-major bleeding (CRNMB) was evaluated and their associated risk factors were identified using univariate and multivariate models. Furthermore, the impact of anticoagulant treatment outcomes on all-cause mortality was analyzed by Cox proportional hazards model and Kaplan-Meier method.

**Results:**

This study included 1,792 CRC patients with cancer-associated VTE. In competing-risk multivariate analysis, independent predictors of recurrent VTE included age (HR with 95%CI: 1.005 [1.002-1.008] per year), history of VTE (4.288 [2.902-6.334]), index pulmonary embolism (PE) (1.698 [1.252-2.303]), ECOG ≥ 2 (1.561 [1.036-2.350]), hemoglobin < 100 g/L (1.363 [1.045-1.778]), and aPTT > 36.5 s (2.034 [1.223-3.383]); whereas recent major surgery or trauma within 1 month (0.451 [0.259-0.786]) and tumor stage II (0.607 [0.377-0.978]) or III (0.562 [0.333-0.949]) were associated with lower recurrence risk. Independent predictors of MB included age ≥ 75 (1.637 [1.011-2.652]), history of MB (5.320 [1.880-15.050]), ECOG ≥ 2 (9.979 [4.292-23.203]), antiplatelet therapy (2.592 [1.539-4.367]), and platelet count < 100×10^9^/L (2.685 [1.336-5.397]); whereas tumor stage III (0.122 [0.053-0.278]) and metastatic cancer (0.190 [0.086-0.421]) predicted lower bleeding risk. Similarly, independent predictors of CRNMB included age ≥ 75 (1.465 [1.005-2.137]), ECOG ≥ 2 (1.750 [1.184-2.586]), hemoglobin < 100 g/L (1.870 [1.316-2.657]), and platelet count < 100×10^9^/L (2.057 [1.076-3.932]). Recurrent VTE, MB, and CRNMB each adversely impacted one-year survival.

**Conclusions:**

The independent risk factors identified in this study may serve as a reference for improving risk stratification in CRC patients receiving anticoagulant treatment. Additionally, adverse outcomes such as VTE recurrence, MB, and CRNMB significantly increase the one-year all-cause mortality risk in CRC patients.

## Introduction

Cancer and venous thromboembolism (VTE), which includes deep vein thrombosis (DVT) and pulmonary embolism (PE), are closely linked by a bidirectional relationship (1). Cancer is responsible for approximately 18% of all VTE cases (2), and cancer-associated VTE accounts for about 20% of the total VTE disease burden (3). Managing VTE in cancer patients is particularly challenging due to the increased risk of thrombus recurrence and bleeding events associated with anticoagulant therapy (4).

The development of cancer-associated VTE involves a complex interplay of multiple overlapping mechanisms. The risk factors contributing to cancer-associated VTE can be classified into categories such as tumor-specific factors, patient-specific characteristics, treatment-related factors, the site of the index VTE, and laboratory findings. The combination of these risk factors increases the chances of recurrent thrombosis and bleeding events, both of which have detrimental effects on survival. It is well-documented that VTE negatively impacts the survival of cancer patients, with those developing PE experiencing a significantly higher mortality rate compared to those without PE (24.8% vs 6.5%, *p*-value < 0.0001) (5, 6). Prior research has also demonstrated that anticoagulant treatment outcomes, including VTE recurrence, major bleeding (MB), and clinically relevant non-major bleeding (CRNMB), can adversely affect patient prognosis (7).

Patients with colorectal cancer (CRC) face an elevated risk of both VTE and MB (8). A subsequent analysis of the Hokusai study indicated that the increased MB risk was particularly significant in patients with gastrointestinal cancers (9). Data from the RIETE registry revealed that patients with gastrointestinal or genitourinary cancers had higher incidences of bleeding, whereas those with brain or lung cancers were more prone to thrombotic events (10). A recent meta-analysis of four studies comparing direct oral anticoagulants (DOACs) with low molecular weight heparin (LMWH) reported a higher bleeding risk associated with DOACs, especially in patients with gastrointestinal cancers (11). However, a subgroup analysis of the Caravaggio trial showed that the incidence of major gastrointestinal bleeding in CRC patients was similar for those treated with apixaban and those treated with LMWH, regardless of cancer type (12). Despite concerns about the bleeding risk posed by DOACs in CRC patients (13), these anticoagulants, including apixaban and rivaroxaban, are commonly used in clinical practice (14, 15). Major trials examining the treatment of cancer-associated VTE with anticoagulants often underrepresent CRC patients. Balancing the risks of thrombosis recurrence and bleeding is challenging and necessitates a nuanced, individualized approach to optimize decision-making for anticoagulant therapy in this population.

To the best of our knowledge, clinical risk factors influencing anticoagulant outcomes in CRC patients have been sparsely systematically investigated in large cohorts. Data on the rates of recurrent thrombosis, MB, and CRNMB in this population remain scarce. Therefore, we conducted a comprehensive retrospective cohort study of CRC-associated VTE patients who received standard anticoagulant treatment according to local guidelines. Our study aimed to evaluate the incidence of recurrent VTE, MB, and CRNMB, and to identify their associated risk factors. Furthermore, we analyzed the impact of anticoagulant treatment outcomes on the all-cause mortality.

## Materials and methods

### Study design and patients

**Figure 1:**
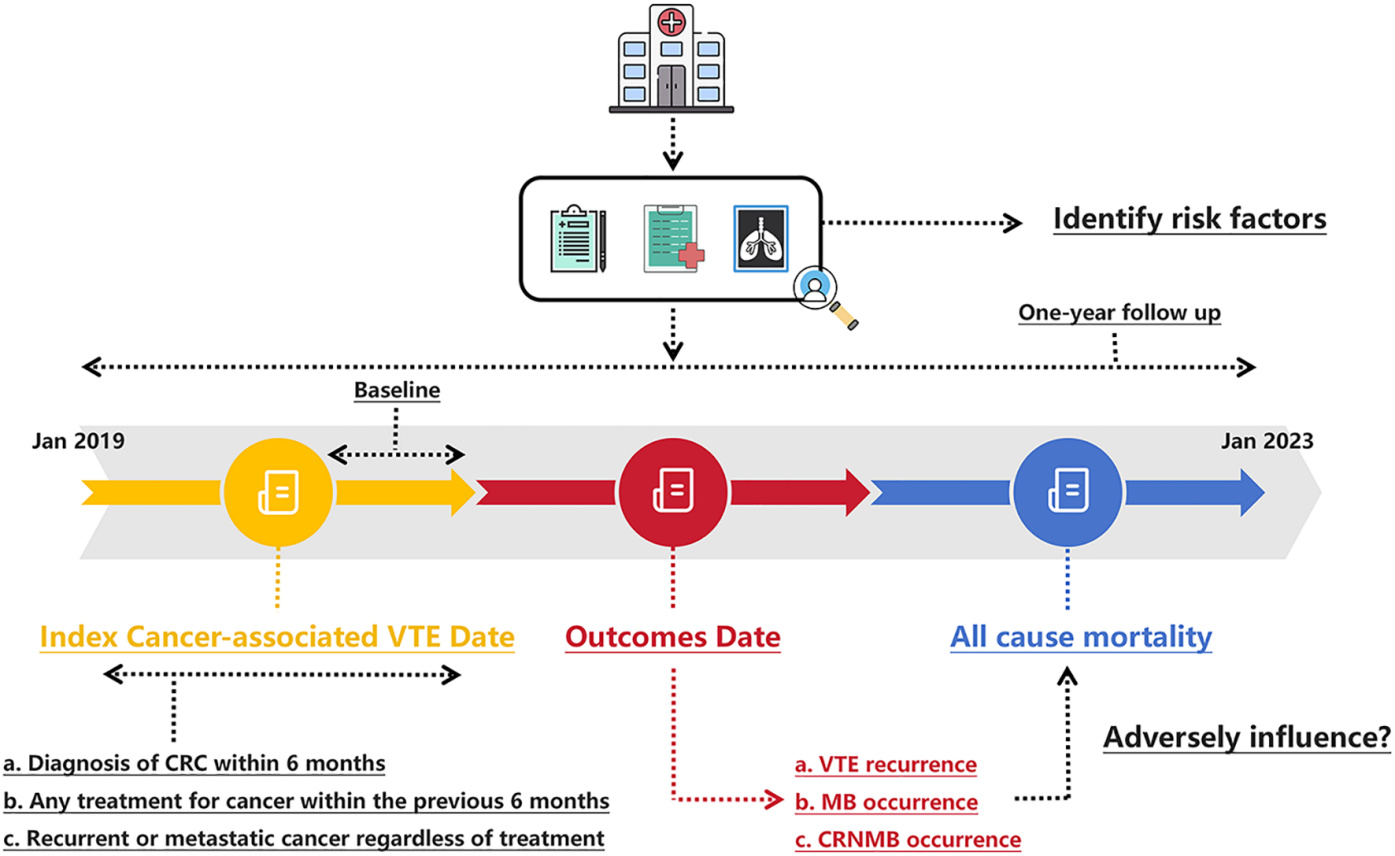
Schematic of the study design. CRC, colorectal cancer; CRNMB, clinical relevant non major bleeding; MB, major bleeding; VTE, venous thromboembolism.

We conducted a single-center retrospective chart review of patients with histologically confirmed CRC and symptomatic or incidental VTE, who received anticoagulant treatment at The Sixth Affiliated Hospital, Sun Yat-sen University from January 2019 to January 2023 (**Figure 1**). Initially, a query was conducted on a prospectively maintained gastrointestinal cancer database to identify all patients with CRC and VTE. Subsequently, each patient’s electronic record was reviewed to determine whether they met the inclusion criteria. This study was registered (NCT06440044) and approved by the Institutional Review Board of the Sixth Affiliated Hospital of Sun Yat-sen University, with a waiver of informed consent granted because of the retrospective nature of the research (approval NO. 2024ZSLYEC-226). The study was conducted in accordance with the Helsinki Declaration (43).

Briefly, CRC patients with VTE who were treated with an anticoagulant (rivaroxaban or LMWH) for at least six months were identified. Patients diagnosed with PE and/or DVT using radiologic imaging techniques, such as CT or ultrasound, were classified as having VTE. There was no standardized protocol or prospective screening plan in place for detecting occult VTE. Both symptomatic individuals diagnosed based on imaging prompted by symptoms, and asymptomatic individuals identified through imaging performed for other medical purposes (such as cancer restaging), were included. VTE was considered cancer-related if the patient had a diagnosis of CRC within six months before or after the VTE diagnosis, any cancer treatment within the previous six months, or recurrent/metastatic cancer. Participation in this study required active anticoagulant treatment. Beyond this, no specific exclusion criteria were applied. Prescription adherence and persistence were verified by examining prescription refill records and provider notes. Cancer treatment at the time of anticoagulant initiation, tumor status, baseline renal function, hemoglobin concentration, global coagulation tests, D-dimer level, and results of other laboratory tests were assessed when available.

### Primary and secondary outcomes

The primary outcome was objectively confirmed recurrent VTE, including DVT and PE. Recurrent DVT was verified using duplex ultrasonography, venography, CT, or MRI. Recurrent PE was confirmed through methods such as CT, MRI, conventional pulmonary angiography, or VQ (ventilation/perfusion) imaging. Incidental VTE recurrences were detected via surveillance imaging. To be classified as a recurrent event, a new filling defect had to be evident on the second study and not appreciated on the original images, or an interval study clearly showing thrombus resolution.

The safety outcome was bleeding events including MB and CRNMB. MB was characterized by overt bleeding accompanied by a hemoglobin drop of ≥ 2 g/dL, the need for transfusion of ≥ 2 units of packed red blood cells, or bleeding in critical areas such as intracranial, intraspinal, intraocular, pericardial, retroperitoneal, or intramuscular regions causing compartment syndrome, or fatal bleeding (16). The secondary safety outcome, CRNMB, was defined as overt bleeding that did not meet the criteria for MB yet required medical intervention, led to unscheduled healthcare visits, caused temporary discontinuation of treatment, or impaired daily activities (17). The objective of this analysis was to determine the risk factors associated with recurrence and bleeding in CRC patients with VTE. All-cause mortality included deaths from any cause, irrespective of the underlying mechanism.

### Statistical analysis

Continuous variables were described as means and standard deviations, while discrete variables were summarized by frequencies and percentages. Univariate analysis was performed to assess the association between each potential predictor and the outcomes: recurrent VTE, MB, and CRNMB. Hazard ratios (HRs) along with their 95% confidence intervals (CIs) were presented. Variables with potential predictive value (univariate *p*-value ≤ 0.05) were further assessed using Cox proportional hazards multivariable analysis, considering recurrent VTE, MB, and CRNMB as the dependent variables. Variables excluded during the model selection process were not reintroduced. Due to the high expected mortality rate among the study patients, Fine and Gray risk-adjusted models were employed to evaluate recurrent VTE and bleeding, with death treated as a competing event. The results were expressed as sub-distribution hazard ratios (sHR) with corresponding 95% CIs and *p*-values.

Actual rates of outcomes, including VTE recurrence, MB, and CRNMB, were estimated using life-table methods. Predicted rates were visualized using cumulative incidence plots, with death considered as a competing event. One-year survival was estimated using the Kaplan-Meier method. The relationship between recurrence, bleeding, and mortality was assessed by incorporating these factors as time-dependent covariates in a Cox proportional hazards model, treating death as the outcome. Study data were collected and managed using Epidata 3.1 (EpiData for Windows; EpiData Association, Odense, Denmark). Data analysis was conducted using SPSS version 20.0 (IBM, Armonk, NY, USA) and the R statistical package (https://cran.r-project.org). All reported *p*-values in this study are two-tailed, with *p*-values < 0.05 considered statistically significant.

## Results

### Patient characteristics at baseline

Over the study period, 1,792 CRC patients with VTE were enrolled (**Supplementary Figure S1**). Among these, 328 (18.3%) experienced recurrent VTE despite anticoagulant therapy, classifying them as anticoagulant failures. Additionally, 51 patients (2.8%) experienced MB associated with the use of anticoagulants, and 125 patients (6.9%) experienced CRNMB. The clinical demographics, including patient-related factors, cancer-related factors, and baseline laboratory results, were stratified based on VTE recurrence (**Table 1**), MB (**Table 2**), and CRNMB (**Table 3**) outcomes. There were no significant differences in these outcomes between patients treated with LMWH and those treated with rivaroxaban.

**Table 1:** Predictors for recurrent VTE at univariate analysis.

Variable	All patients, n = 1792	Recurrent VTE patients, n = 328	Non Recurrent VTE patients, n = 1464	HR (95% CI)	*p*- value	sHR (95% CI)	*p*-value
Patient-related factors							
Age, mean (± SD)	62.3 ± 18.6	63.3 ± 35.1	62.1 ± 12.2	1.00 (0.99-1.01)	0.11	1.01 (1.00-1.01)	0.04
≥ 70, n (%)	496 (27.7)	90 (27.4)	406 (27.7)	1.01 (0.79-1.28)	0.97	1.09 (0.87-1.36)	0.44
≥ 75, n (%)	256 (14.3)	45 (13.7)	211 (14.4)	0.98 (0.71-1.34)	0.89	1.19 (0.90-1.57)	0.22
Female gender, n (%)	775 (43.2)	147 (44.8)	628 (42.9)	1.04 (0.93-1.16)	0.51	1.12 (0.91-1.37)	0.29
BMI, kg/m^2^, mean (± SD)	22.9 ± 3.4	23.4 ± 3.4	22.8 ± 3.4	1.04 (1.01-1.08)	0.01	1.04 (1.01-1.07)	0.01
< 18.5, n (%)	168 (9.4)	18 (5.5)	150 (10.2)	0.54 (0.33-0.87)	0.01	0.69 (0.47-1.03)	0.07
≥ 18.5 and ≤ 23.9, n (%)	955 (53.3)	178 (54.3)	777 (53.1)	1.05 (0.84-1.29)	0.69	0.98 (0.79-1.19)	0.83
> 23.9 and ≤ 28, n (%)	556 (31.0)	102 (31.1)	454 (31.0)	1.01 (0.79-1.27)	0.95	1.02 (0.82-1.27)	0.88
> 28, n (%)	113 (6.3)	30 (9.1)	83 (5.7)	1.54 (1.06-2.24)	0.02	1.53 (1.07-2.17)	0.02
History of VTE, n (%)	40 (2.2)	34 (10.4)	6 (0.4)	6.64 (4.65-9.49)	< 0.01	5.75 (4.03-8.19)	< 0.01
History of major bleeding, n (%)	15 (0.8)	3 (0.9)	12 (0.8)	1.07 (0.34-3.33)	0.91	0.92 (0.29-2.85)	0.88
History of PVT, n (%)	40 (2.2)	6 (1.8)	34 (2.3)	0.81 (0.36-1.81)	0.60	0.82 (0.39-1.73)	0.61
Diagnosis of index VTE							
Index PE (with or without DVT), n (%)	152 (8.5)	50 (15.2)	102 (7.0)	2.13 (1.58-2.88)	< 0.01	2.04 (1.53-2.72)	< 0.01
Isolated DVT							
Isolated distal DVT, n (%)	720 (40.2)	96 (29.3)	624 (42.6)	0.60 (0.47-0.76)	< 0.01	0.64 (0.52-0.80)	< 0.01
Proximal DVT with or without distal DVT, n (%)	920 (51.3)	182 (55.5)	738 (50.4)	1.18 (0.95-1.47)	0.14	1.14 (0.93-1.39)	0.22
Symptomatic VTE, n (%)	1327 (74.1)	236 (72.0)	1091 (74.5)	0.89 (0.70-1.14)	0.37	0.94 (0.75-1.19)	0.62
ECOG performance status ≥ 2, n (%)	1110 (61.9)	231 (70.4)	879 (60.0)	1.49 (1.17-1.88)	< 0.01	1.76 (1.39-2.21)	< 0.01
Major surgery or trauma in previous month, n (%)	174 (9.7)	12 (3.7)	162 (11.1)	0.33 (0.19-0.59)	< 0.01	0.34 (0.19-0.57)	< 0.01
Concomitant antiplatelet therapy, n (%)	143 (8.0)	28 (8.5)	115 (7.9)	1.11 (0.75-1.63)	0.61	1.19 (0.84-1.69)	0.32
Rivaroxaban use, n (%)	479 (26.7)	102 (31.1)	377 (25.8)	1.21 (0.99-1.60)	0.06	1.22 (0.98-1.52)	0.08
CRC-related Factors							
Tumor stage							
Stage I, n (%)	157 (8.8)	29 (8.8)	128 (8.7)	1.02 (0.70-1.49)	0.93	0.97 (0.68-1.39)	0.88
Stage II, n (%)	329 (18.4)	43 (13.1)	286 (19.5)	0.67 (0.48-0.92)	0.02	0.59 (0.43-0.81)	< 0.01
Stage III, n (%)	712 (39.7)	106 (32.3)	606 (41.4)	0.71 (0.56-0.89)	< 0.01	0.73 (0.59-0.91)	< 0.01
Stage IV, n (%)	594 (33.1)	150 (45.7)	444 (30.3)	1.75 (1.41-2.18)	< 0.01	1.82 (1.49-2.23)	< 0.01
Primary tumor site							
Right colon, n (%)	306 (17.1)	53 (16.2)	253 (17.3)	0.93 (0.70-1.25)	0.64	1.12 (0.86-1.45)	0.39
Transverse colon, n (%)	93 (5.2)	22 (6.7)	71 (4.8)	1.32 (0.86-2.04)	0.21	1.19 (0.78-1.82)	0.42
Left colon, n (%)	561 (31.3)	107 (32.6)	454 (31.0)	1.07 (0.85-1.34)	0.59	1.04 (0.84-1.29)	0.71
Rectum, n (%)	832 (46.4)	146 (44.5)	686 (46.9)	0.92 (0.74-1.15)	0.92	0.87 (0.71-1.07)	0.18
Metastatic cancer, n (%)	594 (33.1)	150 (45.7)	444 (30.3)	1.75 (1.41-2.18)	< 0.01	1.82 (1.49-2.23)	< 0.01
Ongoing or recent chemotherapy, n (%)	915 (51.1)	209 (63.7)	706 (48.2)	1.72 (1.37-2.15)	< 0.01	1.58 (1.28-1.94)	< 0.01
CAPEOX^a^, n (%)	150 (8.4)	28 (8.5)	122 (8.3)	1.02 (0.69-1.50)	0.92	1.01 (0.69-1.49)	0.95
FOLFOX^b^, n (%)	623 (34.8)	117 (35.7)	506 (34.6)	1.06 (0.54-1.82)	0.95	1.05 (0.54-1.81)	0.96
FOLFOXIRI^c^, n (%)	207 (11.6)	48 (14.6)	159 (10.9)	1.32 (0.97-1.80)	0.07	1.32 (0.97-1.79)	0.07
Bevacizumab use, n (%)	151 (8.4)	43 (13.1)	108 (7.4)	1.69 (1.23-2.33)	< 0.01	1.65 (1.22-2.23)	< 0.01
Cetuximab use, n (%)	129 (7.2)	31 (9.5)	98 (6.7)	1.37 (0.95-1.99)	0.10	1.37 (0.95-1.98)	0.10
Other targeted therapies, n (%)	42 (2.3)	11 (3.4)	31 (2.1)	1.51 (0.83-2.76)	0.18	1.49 (0.82-2.73)	0.19
Ongoing or recent radiotherapy, n (%)	113 (6.3)	23 (7.0)	90 (6.1)	1.11 (0.73-1.71)	0.62	1.19 (0.81-1.76)	0.37
Laboratory results							
RBC counts, m/μL, mean (± SD)	3.8 ± 1.1	3.8 ± 2.0	3.8 ± 0.6	1.03 (0.91-1.17)	0.65	0.94 (0.79-1.10)	0.42
WBC counts, K/μL, mean (± SD)	10.2 ± 2.9	9.9 ± 3.1	10.3 ± 2.9	0.99 (0.98-1.01)	0.48	1.00 (0.99-1.01)	0.72
> 11, n (%)	923 (51.5)	154 (47.0)	769 (52.5)	0.83 (0.67-1.03)	0.09	0.87 (0.71-1.06)	0.17
Platelet counts, K/μL, mean (± SD)	232.4 ± 83.9	233.6 ± 83.3	232.1 ± 84.0	1.00 (0.99-1.01)	0.79	1.00 (0.99-1.01)	0.24
< 100, n (%)	47 (2.6)	9 (2.7)	38 (2.6)	1.04 (0.54-2.02)	0.91	1.48 (0.87-2.52)	0.15
Hemoglobin, g/L, mean (± SD)	107.1 ± 16.9	103.9 ± 19.3	107.7 ± 16.3	0.99 (0.98-1.01)	0.06	0.99 (0.98-0.99)	< 0.01
< 100, n (%)	376 (21.1)	97 (29.6)	279 (19.1)	1.66 (1.31-2.11)	< 0.01	1.82 (1.46-2.26)	< 0.01
CRP, mg/L, mean (± SD)	53.1 ± 5.9	57.5 ± 6.2	52.2 ± 5.9	1.00 (1.00-1.01)	0.06	1.00 (1.00-1.01)	< 0.01
> 10, n (%)	1632 (91.1)	301 (91.8)	1331 (90.9)	1.12 (0.76-1.67)	0.56	1.13 (0.78-1.63)	0.51
Creatinine clearance, mL/min, mean (± SD)	81.5 ± 23.1	83.9 ± 29.6	80.9 ± 21.3	1.01 (1.00-1.01)	0.04	1.00 (0.99-1.01)	0.09
≥ 90, n (%)	472 (26.3)	103 (31.4)	369 (25.2)	1.29 (1.03-1.64)	0.03	1.24 (0.99-1.55)	0.05
≥ 60 and < 90, n (%)	1102 (61.5)	179 (54.6)	923 (63.0)	0.73 (0.59-0.91)	< 0.01	0.77 (0.63-0.94)	0.01
≥ 30 and < 60, n (%)	195 (10.9)	39 (11.9)	156 (10.7)	1.13 (0.81-1.59)	0.46	1.14 (0.83-1.55)	0.42
< 30, n (%)	23 (1.3)	7 (2.1)	16 (1.1)	1.74 (0.82-3.68)	0.15	1.49 (0.71-3.17)	0.29
PT, sec, mean (± SD)	12.3 ± 2.6	12.7 ± 2.7	12.2 ± 2.6	1.02 (1.01-1.04)	0.03	1.05 (1.03-1.06)	< 0.01
> 12.5, n (%)	336 (18.8)	83 (25.3)	253 (17.3)	1.52 (1.18-1.95)	< 0.01	1.59 (1.27-2.01)	< 0.01
aPTT, sec, mean (± SD)	30.2 ± 4.1	30.5 ± 5.7	29.9 ± 3.5	1.02 (1.01-1.04)	< 0.01	1.04 (1.02-1.05)	< 0.01
> 36.5, n (%)	40 (2.2)	16 (4.9)	24 (1.6)	2.63 (1.59-4.35)	< 0.01	2.71 (1.69-4.35)	< 0.01
INR, mean (± SD)	1.1 ± 0.2	1.1 ± 0.2	1.1 ± 0.2	1.28 (1.05-1.56)	0.02	1.67 (1.42-1.96)	< 0.01
> 1.2, n (%)	151 (8.4)	46 (14.0)	105 (7.2)	1.87 (1.37-2.55)	< 0.01	1.87 (1.39-2.50)	< 0.01
Fibrinogen, g/L, mean (± SD)	3.4 ± 0.7	3.4 ± 0.8	3.4 ± 0.6	1.03 (0.87-1.21)	0.75	1.05 (0.90-1.22)	0.51
< 2.00, n (%)	35 (2.0)	9 (2.7)	26 (1.8)	1.49 (0.77-2.91)	0.23	1.67 (0.92-3.05)	0.09
Fibrin D-dimer, mg/L, mean (± SD)	3.6 ± 2.5	3.8 ± 2.5	3.6 ± 2.6	1.03 (0.99-1.07)	0.13	1.04 (1.01-1.08)	0.04
> 1.2, n (%)	1536 (85.7)	286 (87.2)	1250 (85.4)	1.16 (0.84-1.61)	0.37	1.27 (0.93-1.73)	0.14
Score for recurrent VTE risk in cancer-associated thrombosis							
Ottawa score							
≥ 1, n (%)	725 (40.5)	149 (45.4)	576 (39.3)	1.24 (0.99-1.54)	0.06	1.25 (1.02-1.53)	0.03
< 1, n (%)	1067 (59.5)	179 (54.6)	888 (60.7)	–	–	–	–

aPTT, activated partial thromboplastin time; BMI, body mass index; CI, confidence interval; CRC, colorectal cancer; CRP, C-reactive protein; DVT, deep venous thrombosis; ECOG, Eastern Cooperative Oncology Group; HR, hazard ratio; INR, international normalized ratio; PE, pulmonary embolism; PT, prothrombin time; PVT, portal venous thrombosis; RBC, red blood cell; sHR, sub-distribution hazard ratio; SD, standard deviation; VTE, venous thromboembolism; WBC, white blood cell.

Percentages are based on the total number of patients in each modified intention-to-treat (mITT) stratum.

Death is considered as competing risk in sHR calculation.

The univariate model employed a significance level of 0.05.

HR and sHR, along with their corresponding 95% CIs and *p*-values, were derived from a Cox proportional hazards univariate model, using only the predictor as the model’s covariate.

a cycle of CAPEOX consists of the following: capecitabine 1000 mg/m^2^ p.o. twice daily for 14 out of 21 days.

b A cycle of FOLFOX consists of the following: Day 1: i.v. oxaliplatin 85 mg/m^2^ in 120 min; i.v. leucovorin 200 mg/m^2^ in 2 h; 5-FU 400 mg/m^2^ bolus i.v. then 2400 mg/m^2^ perfusion i.v. over 46 h; The next chemotherapy cycle was repeated on the 15th day.

c A cycle of FOLFOXIRI consists of the following: Day 1: i.v. irinotecan 165 mg/m^2^ in 90 min; i.v. oxaliplatin 85 mg/m^2^ in 120 min; i.v. leucovorin 200 mg/m^2^ in 2 h; 5-FU 400 mg/m^2^ bolus i.v. then 2400 mg/m^2^ perfusion i.v. over 46 h; The next chemotherapy cycle was repeated on the 15th day.

**Table 2:** Predictors for MB at univariate analysis.

Variable	All patients, n = 1792	MB patients, n = 51	Non MB patients, n = 1741	HR (95% CI)	*p*-value	sHR (95% CI)	*p*-value
Patient-related Factors							
Age, mean (± SD)	62.3 ± 18.6	63.4 ± 12.9	62.2 ± 18.8	1.00 (0.99-1.01)	0.68	1.00 (0.99-1.01)	0.41
≥ 70, n (%)	496 (27.7)	17 (33.3)	479 (27.5)	1.31 (0.73-2.35)	0.36	1.39 (0.94-2.07)	0.09
≥ 75, n (%)	256 (14.3)	10 (19.6)	246 (14.1)	1.47 (0.74-2.94)	0.27	1.71 (1.09-2.69)	0.02
Female gender, n (%)	775 (43.2)	23 (45.1)	752 (43.2)	1.08 (0.62-1.87)	0.79	1.18 (0.81-1.72)	0.38
BMI, kg/m^2^, mean (± SD)	22.9 ± 3.4	22.6 ± 4.3	22.9 ± 3.4	0.97 (0.89-1.05)	0.44	1.01 (0.95-1.06)	0.86
< 18.5, n (%)	168 (9.4)	7 (13.7)	161 (9.2)	1.56 (0.70-3.46)	0.28	1.44 (0.82-2.52)	0.20
≥ 18.5 and ≤ 23.9, n (%)	955 (53.3)	25 (49.0)	930 (53.4)	0.84 (0.48-1.45)	0.53	0.75 (0.52-1.09)	0.14
> 23.9 and ≤ 28, n (%)	556 (31.0)	16 (31.4)	540 (31.0)	1.02 (0.56-1.84)	0.95	1.17 (0.79-1.74)	0.42
> 28, n (%)	113 (6.3)	3 (5.9)	110 (6.3)	0.93 (0.29-2.98)	0.90	1.01 (0.47-2.18)	0.98
History of VTE, n (%)	40 (2.2)	1 (2.0)	39 (2.2)	0.88 (0.12-6.35)	0.89	0.81 (0.19-3.27)	0.76
History of major bleeding, n (%)	15 (0.8)	3 (5.9)	12 (0.7)	8.05 (2.51-25.85)	< 0.01	5.18 (1.91-14.05)	< 0.01
History of PVT, n (%)	40 (2.2)	3 (5.9)	37 (2.1)	2.74 (0.85-8.79)	0.09	1.69 (0.63-4.61)	0.30
Diagnosis of index VTE							
Index PE (with or without DVT), n (%)	152 (8.5)	10 (19.6)	142 (8.2)	2.71 (1.36-5.40)	< 0.01	1.91 (1.12-3.24)	0.02
Isolated DVT, n (%)							
Isolated distal DVT, n (%)	720 (40.2)	19 (37.3)	701 (40.3)	0.88 (0.50-1.56)	0.67	0.82 (0.55-1.21)	0.31
Proximal DVT with or without distal DVT, n (%)	920 (51.3)	22 (43.1)	898 (51.6)	0.71 (0.41-1.24)	0.23	0.94 (0.65-1.37)	0.74
Symptomatic VTE, n (%)	1327 (74.1)	38 (74.5)	1289 (74.0)	1.03 (0.55-1.93)	0.94	1.33 (0.84-2.11)	0.22
ECOG performance status ≥ 2, n (%)	1110 (61.9)	33 (64.7)	1077 (61.9)	1.13 (0.64-2.01)	0.68	2.49 (1.56-3.98)	< 0.01
Major surgery or trauma in previous month, n (%)	174 (9.7)	2 (3.9)	172 (9.9)	0.38 (0.09-1.55)	0.18	0.34 (0.13-0.93)	0.04
Concomitant antiplatelet therapy, n (%)	143 (8.0)	14 (27.5)	129 (7.4)	4.52 (2.44-8.36)	< 0.01	2.73 (1.68-4.43)	< 0.01
Rivaroxaban use, n (%)	479 (26.7)	16 (31.4)	463 (26.6)	1.26 (0.69-2.27)	0.45	1.03 (0.68-1.57)	0.89
CRC-related Factors							
Tumor stage							
Stage I, n (%)	157 (8.8)	6 (11.8)	151 (8.7)	1.41 (0.60-3.29)	0.43	1.05 (0.55-2.02)	0.87
Stage II, n (%)	329 (18.4)	14 (27.5)	315 (18.1)	1.69 (0.92-3.13)	0.09	0.87 (0.53-1.45)	0.59
Stage III, n (%)	712 (39.7)	7 (13.7)	705 (40.5)	0.24 (0.11-0.53)	< 0.01	0.48 (0.31-0.74)	< 0.01
Stage IV, n (%)	594 (33.1)	24 (47.1)	570 (32.7)	1.81 (1.04-3.13)	0.04	2.06 (1.42-2.99)	< 0.01
Primary tumor site							
Right colon, n (%)	306 (17.1)	15 (29.4)	291 (16.7)	2.06 (1.13-3.75)	0.02	1.87 (1.23-2.85)	< 0.01
Transverse colon, n (%)	93 (5.2)	5 (9.8)	88 (5.1)	2.00 (0.79-5.04)	0.14	1.26 (0.59-2.71)	0.55
Left colon, n (%)	561 (31.3)	12 (23.5)	549 (31.5)	0.67 (0.35-1.28)	0.23	0.86 (0.57-1.29)	0.47
Rectum, n (%)	832 (46.4)	19 (37.3)	813 (46.7)	0.68 (0.39-1.20)	0.18	0.70 (0.48-1.03)	0.07
Metastatic cancer, n (%)	594 (33.1)	24 (47.1)	570 (32.7)	1.81 (1.04-3.13)	0.04	2.06 (1.42-2.99)	< 0.01
Ongoing or recent chemotherapy, n (%)	915 (51.1)	19 (37.3)	896 (51.5)	0.56 (0.32-0.99)	0.05	0.85 (0.58-1.23)	0.39
CAPEOX^a^, n (%)	150 (8.4)	5 (9.8)	145 (8.3)	1.20 (0.48-3.02)	0.69	1.19 (0.48-3.02)	0.70
FOLFOX^b^, n (%)	623 (34.8)	16 (31.4)	607 (34.9)	0.66 (0.42-1.00)	0.05	0.68 (0.43-1.01)	0.06
FOLFOXIRI^c^, n (%)	207 (11.6)	5 (9.8)	202 (11.6)	0.83 (0.33-2.09)	0.69	1.02 (0.43-2.38)	0.97
Bevacizumab use, n (%)	151 (8.4)	5 (9.8)	146 (8.4)	1.19 (0.47-2.99)	0.72	1.75 (1.01-3.01)	0.04
Cetuximab use, n (%)	129 (7.2)	3 (5.9)	126 (7.2)	0.80 (0.25-2.57)	0.71	0.79 (0.25-2.56)	0.70
Other targeted therapies, n (%)	42 (2.3)	4 (7.8)	38 (2.2)	2.61 (0.64-6.78)	0.13	2.66 (0.83-8.55)	0.10
Ongoing or recent radiotherapy, n (%)	113 (6.3)	0	113 (6.5)	0.05 (0.00-6.45)	0.22	1.00 (0.47-2.15)	0.99
Laboratory results							
RBC counts, m/μL, mean (± SD)	3.8 ± 1.1	3.9 ± 3.6	3.8 ± 0.9	1.09 (0.96-1.24)	0.17	0.89 (0.65-1.21)	0.44
WBC counts, K/μL, mean (± SD)	10.2 ± 2.9	12.1 ± 3.2	10.2 ± 2.9	1.02 (0.99-1.05)	0.12	1.02 (0.99-1.03)	0.13
> 11, n (%)	923 (51.5)	28 (54.9)	895 (51.4)	1.15 (0.66-1.99)	0.62	0.88 (0.61-1.28)	0.49
Platelet counts, K/μL, mean (± SD)	232.4 ± 83.9	232.3 ± 112.8	232.4 ± 82.9	1.00 (0.99-1.00)	0.99	1.00 (1.00-1.01)	0.06
< 100, n (%)	47 (2.6)	4 (7.8)	43 (2.5)	3.21 (1.15-8.89)	0.03	4.44 (2.38-8.27)	< 0.01
Hemoglobin, g/L, mean (± SD)	107.1 ± 16.9	96.7 ± 24.6	107.3 ± 16.6	0.97 (0.96-0.98)	< 0.01	0.97 (0.96-0.98)	< 0.01
< 100, n (%)	376 (21.1)	21 (41.2)	355 (20.4)	2.68 (1.54-4.68)	< 0.01	2.93 (2.01-4.27)	< 0.01
CRP, mg/L, mean (± SD)	53.1 ± 5.9	72.5 ± 7.4	52.6 ± 5.9	1.01 (1.01-1.02)	< 0.01	1.01 (1.00-1.01)	< 0.01
> 10, n (%)	1632 (91.1)	46 (90.2)	1586 (91.1)	0.90 (0.36-2.28)	0.83	1.26 (0.61-2.58)	0.54
Creatinine clearance, mL/min, mean (± SD)	81.5 ± 23.1	76.7 ± 26.7	81.6 ± 22.9	0.99 (0.98-1.00)	0.12	0.99 (0.98-1.00)	0.29
≥ 90, n (%)	472 (26.3)	13 (25.5)	459 (26.4)	0.96 (0.51-1.80)	0.90	1.05 (0.69-1.59)	0.82
≥ 60 and < 90, n (%)	1102 (61.5)	27 (52.9)	1075 (61.7)	0.69 (0.40-1.21)	0.19	0.80 (0.55-1.17)	0.25
≥ 30 and < 60, n (%)	195 (10.9)	8 (15.7)	187 (10.7)	1.54 (0.72-3.27)	0.26	1.31 (0.76-2.25)	0.33
< 30, n (%)	23 (1.3)	3 (5.9)	20 (1.1)	5.21 (1.62-16.72)	< 0.01	2.35 (0.75-7.39)	0.15
PT, sec, mean (± SD)	12.3 ± 2.6	14.4 ± 12.5	12.3 ± 1.6	1.06 (1.04-1.08)	< 0.01	1.10 (1.06-1.14)	< 0.01
> 12.5, n (%)	336 (18.8)	14 (27.5)	322 (18.5)	1.66 (0.89-3.07)	0.11	1.99 (1.33-2.98)	< 0.01
aPTT, sec, mean (± SD)	30.2 ± 4.1	34.5 ± 17.9	29.9 ± 2.6	1.05 (1.04-1.07)	< 0.01	1.06 (1.04-1.07)	< 0.01
> 36.5, n (%)	40 (2.2)	4 (7.8)	36 (2.1)	3.88 (1.39-10.77)	< 0.01	3.79 (1.85-7.78)	< 0.01
INR, mean (± SD)	1.1 ± 0.2	1.3 ± 1.1	1.1 ± 0.2	1.86 (1.47-2.36)	< 0.01	2.99 (1.99-4.48)	< 0.01
> 1.2, n (%)	151 (8.4)	8 (15.7)	143 (8.2)	2.06 (0.97-4.38)	0.06	2.21 (1.33-3.66)	< 0.01
Fibrinogen, g/L, mean (± SD)	3.4 ± 0.7	3.2 ± 0.9	3.4 ± 0.7	0.61 (0.39-0.96)	0.03	0.93 (0.69-1.25)	0.61
< 2.00, n (%)	35 (2.0)	3 (5.9)	32 (1.8)	3.20 (0.99-10.27)	0.06	2.01 (0.74-5.46)	0.17
Fibrin D-dimer, mg/L, mean (± SD)	3.6 ± 2.5	4.4 ± 3.2	3.6 ± 2.5	1.09 (1.01-1.19)	0.03	1.11 (1.05-1.17)	< 0.01
> 1.2, n (%)	1536 (85.7)	44 (86.3)	1492 (85.7)	1.05 (0.47-2.33)	0.90	1.51 (0.81-2.82)	0.19
Bleeding risk factors* ≥ 1, n (%)	1439 (80.3)	41 (80.4)	1398 (80.3)	1.01 (0.50-2.01)	0.99	0.74 (0.37-1.51)	0.41
Bleeding risk factors* ≥ 2, n (%)	321 (17.9)	11 (21.6)	310 (17.8)	1.27 (0.65-2.47)	0.49	0.67 (0.33-1.35)	0.26
Bleeding risk factors* ≥ 3, n (%)	14 (0.8)	1 (0.8)	13 (0.8)	0.04 (0.01-6.84)	0.22	0.04 (0.01-6.51)	0.21

aPTT, activated partial thromboplastin time; BMI, body mass index; CI, confidence interval; CRC, colorectal cancer; CRP, C-reactive protein; DVT, deep venous thrombosis; ECOG, Eastern Cooperative Oncology Group; HR, hazard ratio; INR, international normalized ratio; MB, major bleeding; PE, pulmonary embolism; PT, prothrombin time; PVT, portal venous thrombosis; RBC, red blood cell; sHR, sub-distribution hazard ratio; SD, standard deviation; VTE, venous thromboembolism; WBC, white blood cell.

Percentages are based on the total number of patients in each modified intention-to-treat (mITT) stratum.

Death is considered as competing risk in sHR calculation.

The univariate model employed a significance level of 0.05.

HR and sHR, along with their corresponding 95% CIs and *p*-values, were derived from a Cox proportional hazards univariate model, using only the predictor as the model’s covariate.

*The following bleeding risk factors were considered:

-Major surgery or trauma in previous month.

-Concomitant antiplatelet therapy.

-Advanced or metastatic cancer.

-Use of Bevacizumab.

a cycle of CAPEOX consists of the following: capecitabine 1000 mg/m^2^ p.o. twice daily for 14 out of 21 days.

b A cycle of FOLFOX consists of the following: Day 1: i.v. oxaliplatin 85 mg/m^2^ in 120 min; i.v. leucovorin 200 mg/m^2^ in 2 h; 5-FU 400 mg/m^2^ bolus i.v. then 2400 mg/m^2^ perfusion i.v. over 46 h; The next chemotherapy cycle was repeated on the 15th day.

c A cycle of FOLFOXIRI consists of the following: Day 1: i.v. irinotecan 165 mg/m^2^ in 90 min; i.v. oxaliplatin 85 mg/m^2^ in 120 min; i.v. leucovorin 200 mg/m^2^ in 2 h; 5-FU 400 mg/m^2^ bolus i.v. then 2400 mg/m^2^ perfusion i.v. over 46 h; The next chemotherapy cycle was repeated on the 15th day.

**Table 3:** Predictors for CRNMB at univariate analysis.

Variable	All patients, n = 1792	CRNMB patients, n = 125	Non CRNMB patients, n = 1667	HR (95% CI)	*p*-value	sHR (95% CI)	*p*-value
Patient-related factors							
Age, mean (± SD)	62.3 ± 18.6	63.6 ± 12.3	62.2 ± 19.0	1.00 (0.99-1.01)	0.78	1.00 (0.99-1.01)	0.45
≥ 70, n (%)	496 (27.7)	39 (31.2)	457 (27.4)	1.02 (0.69-1.50)	0.93	1.20 (0.88-1.65)	0.25
≥ 75, n (%)	256 (14.3)	22 (17.6)	234 (14.0)	1.23 (0.77-1.96)	0.39	1.51 (1.04-2.18)	0.03
Female gender, n (%)	775 (43.2)	57 (45.6)	718 (43.1)	1.11 (0.78-1.58)	0.57	1.14 (0.85-1.54)	0.37
BMI, kg/m^2^, mean (± SD)	22.9 ± 3.4	22.5 ± 3.1	22.9 ± 3.4	0.94 (0.89-0.99)	0.02	0.97 (0.93-1.01)	0.14
< 18.5, n (%)	168 (9.4)	14 (11.2)	154 (9.2)	1.71 (1.04-2.81)	0.04	1.58 (1.03-2.44)	0.04
≥ 18.5 and ≤ 23.9, n (%)	955 (53.3)	70 (56.0)	885 (53.1)	1.24 (0.87-1.76)	0.24	1.04 (0.78-1.39)	0.79
> 23.9 and ≤ 28, n (%)	556 (31.0)	37 (29.6)	519 (31.1)	0.60 (0.39-0.92)	0.02	0.79 (0.57-1.11)	0.17
> 28, n (%)	113 (6.3)	4 (3.2)	109 (6.5)	0.87 (0.41-1.86)	0.72	0.87 (0.46-1.65)	0.67
History of VTE, n (%)	40 (2.2)	1 (0.8)	39 (2.3)	0.71 (0.17-2.85)	0.62	0.73 (0.23-2.29)	0.59
History of major bleeding, n (%)	15 (0.8)	0	15 (0.9)	1.94 (0.48-7.85)	0.35	2.06 (0.66-6.44)	0.22
History of PVT, n (%)	40 (2.2)	4 (3.2)	36 (2.2)	1.49 (0.55-4.04)	0.43	1.29 (0.53-3.14)	0.58
Diagnosis of index VTE							
Index PE (with or without DVT), n (%)	152 (8.5)	15 (12.0)	137 (8.2)	0.73 (0.36-1.50)	0.39	0.91 (0.53-1.58)	0.75
Isolated DVT, n (%)							
Isolated distal DVT, n (%)	720 (40.2)	54 (43.2)	666 (40.0)	1.39 (0.98-1.97)	0.07	1.22 (0.91-1.64)	0.18
Proximal DVT with or without distal DVT, n (%)	920 (51.3)	56 (44.8)	864 (51.8)	0.79 (0.56-1.12)	0.19	0.85 (0.63-1.13)	0.26
Symptomatic VTE, n (%)	1327 (74.1)	95 (76.0)	1232 (73.9)	1.06 (0.71-1.59)	0.77	1.22 (0.86-1.74)	0.26
ECOG performance status ≥ 2, n (%)	1110 (61.9)	74 (59.2)	1036 (62.1)	1.37 (0.94-1.99)	0.11	1.93 (1.37-2.71)	< 0.01
Major surgery or trauma in previous month, n (%)	174 (9.7)	12 (9.6)	162 (9.7)	1.18 (0.68-2.06)	0.56	0.85 (0.50-1.44)	0.55
Concomitant antiplatelet therapy, n (%)	143 (8.0)	12 (9.6)	131 (7.9)	0.79 (0.39-1.62)	0.52	1.07 (0.63-1.81)	0.82
Rivaroxaban use, n (%)	479 (26.7)	21 (16.8)	458 (27.5)	0.79 (0.52-1.19)	0.26	0.83 (0.59-1.17)	0.29
CRC-related Factors							
Tumor stage							
Stage I, n (%)	157 (8.8)	11 (8.8)	146 (8.8)	0.62 (0.29-1.32)	0.21	0.75 (0.42-1.34)	0.33
Stage II, n (%)	329 (18.4)	27 (21.6)	302 (18.1)	1.34 (0.89-2.04)	0.16	0.97 (0.66-1.42)	0.86
Stage III, n (%)	712 (39.7)	46 (36.8)	666 (40.0)	0.85 (0.59-1.22)	0.38	0.78 (0.57-1.06)	0.11
Stage IV, n (%)	594 (33.1)	41 (32.8)	553 (33.2)	1.10 (0.76-1.59)	0.60	1.44 (1.07-1.94)	0.02
Primary tumor site							
Right colon, n (%)	306 (17.1)	15 (12.0)	291 (17.5)	1.22 (0.79-1.89)	0.37	1.34 (0.94-1.92)	0.11
Transverse colon, n (%)	93 (5.2)	12 (9.6)	81 (4.9)	1.08 (0.51-2.32)	0.84	0.97 (0.49-1.89)	0.92
Left colon, n (%)	561 (31.3)	47 (37.6)	514 (30.8)	1.07 (0.74-1.56)	0.72	1.02 (0.75-1.41)	0.88
Rectum, n (%)	832 (46.4)	51 (40.8)	781 (46.9)	0.82 (0.57-1.17)	0.27	0.82 (0.61-1.11)	0.19
Metastatic cancer, n (%)	594 (33.1)	41 (32.8)	553 (33.2)	1.10 (0.76-1.59)	0.60	1.44 (1.07-1.94)	0.02
Ongoing or recent chemotherapy, n (%)	915 (51.1)	55 (44.0)	860 (51.6)	0.77 (0.54-1.09)	0.14	0.85 (0.64-1.14)	0.29
CAPEOX^a^, n (%)	150 (8.4)	5 (4.0)	145 (8.7)	0.45 (0.18-1.10)	0.08	0.45 (0.18-1.09)	0.08
FOLFOX^b^, n (%)	623 (34.8)	41 (32.8)	582 (34.9)	0.91 (0.63-1.32)	0.63	0.91 (0.63-1.32)	0.63
FOLFOXIRI^c^, n (%)	207 (11.6)	12 (9.6)	195 (11.7)	0.81 (0.44-1.46)	0.48	0.80 (0.44-1.45)	0.47
Bevacizumab use, n (%)	151 (8.4)	17 (13.6)	134 (8.0)	1.04 (0.56-1.94)	0.89	1.38 (0.86-2.19)	0.18
Cetuximab use, n (%)	129 (7.2)	13 (10.4)	116 (7.0)	1.54 (0.87-2.73)	0.14	1.53 (0.86-2.71)	0.15
Other targeted therapies, n (%)	42 (2.3)	6 (4.8)	36 (2.2)	2.22 (0.98-5.05)	0.06	2.22 (0.98-5.04)	0.06
Ongoing or recent radiotherapy, n (%)	113 (6.3)	9 (7.2)	104 (6.2)	1.03 (0.51-2.11)	0.93	1.39 (0.82-2.37)	0.22
Laboratory results							
RBC counts, m/μL, mean (± SD)	3.8 ± 1.1	3.7 ± 0.5	3.8 ± 1.1	0.92 (0.69-1.20)	0.52	0.81 (0.61-1.01)	0.06
WBC counts, K/μL, mean (± SD)	10.2 ± 2.9	9.6 ± 2.9	10.3 ± 2.9	1.02 (0.99-1.03)	0.10	1.02 (1.00-1.03)	0.04
> 11, n (%)	923 (51.5)	65 (52.0)	858 (51.5)	0.93 (0.65-1.32)	0.68	0.92 (0.68-1.23)	0.56
Platelet counts, K/μL, mean (± SD)	232.4 ± 83.9	226.3 ± 68.8	232.9 ± 84.9	0.99 (0.99-1.00)	0.11	1.00 (0.99-1.01)	0.38
< 100, n (%)	47 (2.6)	2 (1.6)	45 (2.7)	3.49 (1.83-6.67)	< 0.01	3.29 (1.88-5.79)	< 0.01
Hemoglobin, g/L, mean (± SD)	107.1 ± 16.9	108.1 ± 15.8	106.9 ± 17.0	0.98 (0.97-0.99)	< 0.01	0.98 (0.97-0.99)	< 0.01
< 100, n (%)	376 (21.1)	21 (16.8)	355 (21.3)	2.27 (1.57-3.26)	< 0.01	2.37 (1.75-3.20)	< 0.01
CRP, mg/L, mean (± SD)	53.1 ± 5.9	50.2 ± 5.8	53.4 ± 5.9	1.01 (1.00-1.01)	0.03	1.01 (1.00-1.01)	< 0.01
> 10, n (%)	1632 (91.1)	107 (85.6)	1525 (91.5)	1.99 (0.88-4.52)	0.10	1.71 (0.90-3.24)	0.10
Creatinine clearance, mL/min, mean (± SD)	81.5 ± 23.1	78.1 ± 17.6	81.7 ± 23.4	0.99 (0.98-1.01)	0.24	1.00 (0.99-1.01)	0.29
≥ 90, n (%)	472 (26.3)	25 (20.0)	447 (26.8)	0.59 (0.37-0.93)	0.02	0.71 (0.49-1.02)	0.06
≥ 60 and < 90, n (%)	1102 (61.5)	85 (68.0)	1017 (61.0)	1.15 (0.80-1.67)	0.44	1.11 (0.82-1.51)	0.50
≥ 30 and < 60, n (%)	195 (10.9)	14 (11.2)	181 (10.9)	1.33 (0.79-2.21)	0.28	1.23 (0.79-1.91)	0.35
< 30, n (%)	23 (1.3)	1 (0.8)	22 (1.3)	3.46 (1.41-8.46)	0.01	2.39 (0.99-5.83)	0.06
PT, sec, mean (± SD)	12.3 ± 2.6	12.4 ± 2.2	12.3 ± 2.7	1.06 (1.04-1.09)	< 0.01	1.08 (1.05-1.11)	< 0.01
> 12.5, n (%)	336 (18.8)	28 (22.4)	308 (18.5)	1.14 (0.74-1.75)	0.56	1.38 (0.98-1.95)	0.06
aPTT, sec, mean (± SD)	30.2 ± 4.1	29.9 ± 3.6	30.0 ± 4.1	1.05 (1.03-1.06)	< 0.01	1.06 (1.04-1.09)	< 0.01
> 36.5, n (%)	40 (2.2)	3 (2.4)	37 (2.2)	1.86 (0.76-4.56)	0.17	1.90 (0.89-4.05)	0.10
INR, mean (± SD)	1.1 ± 0.2	1.1 ± 0.2	1.1 ± 0.2	1.97 (1.49-2.59)	< 0.01	2.32 (1.70-3.16)	< 0.01
> 1.2, n (%)	151 (8.4)	12 (9.6)	139 (8.3)	1.61 (0.95-2.72)	0.08	1.74 (1.13-2.67)	0.01
Fibrinogen, g/L, mean (± SD)	3.4 ± 0.7	3.4 ± 0.7	3.4 ± 0.7	0.88 (0.66-1.16)	0.36	1.01 (0.80-1.26)	0.96
< 2.00, n (%)	35 (2.0)	3 (2.4)	32 (1.9)	2.70 (1.19-6.13)	0.02	2.29 (1.07-4.87)	0.03
Fibrin D-dimer, mg/L, mean (± SD)	3.6 ± 2.5	3.4 ± 2.6	3.6 ± 2.5	1.08 (1.02-1.14)	0.01	1.09 (1.05-1.14)	< 0.01
> 1.2, n (%)	1536 (85.7)	105 (84.0)	1431 (85.8)	0.87 (0.54-1.40)	0.57	1.13 (0.73-1.75)	0.58
Bleeding risk factors* ≥ 1, n (%)	1439 (80.3)	99 (79.2)	1340 (80.4)	0.94 (0.61-1.45)	0.78	2.83 (0.66-9.98)	0.16
Bleeding risk factors* ≥ 2, n (%)	321 (17.9)	22 (17.6)	299 (17.9)	0.98 (0.62-1.55)	0.92	1.31 (0.51-3.36)	0.57
Bleeding risk factors* ≥ 3, n (%)	14 (0.8)	1 (0.8)	13 (0.8)	1.02 (0.14-7.29)	0.99	0.05 (0.01-9.66)	0.62

aPTT, activated partial thromboplastin time; BMI, body mass index; CI, confidence interval; CRC, colorectal cancer; CRNMB, clinical relevant non major bleeding; CRP, C-reactive protein; DVT, deep venous thrombosis; ECOG, Eastern Cooperative Oncology Group; HR, hazard ratio; INR, international normalized ratio; PE, pulmonary embolism; PT, prothrombin time; PVT, portal venous thrombosis; RBC, red blood cell; sHR, sub-distribution hazard ratio; SD, standard deviation; VTE, venous thromboembolism; WBC, white blood cell.

Percentages are based on the total number of patients in each modified intention-to-treat (mITT) stratum.

Death is considered as competing risk in sHR calculation.

The univariate model employed a significance level of 0.05.

HR and sHR, along with their corresponding 95% CIs and *p*-values, were derived from a Cox proportional hazards univariate model, using only the predictor as the model’s covariate.

*The following bleeding risk factors were considered:

-Major surgery or trauma in previous month.

-Concomitant antiplatelet therapy.

-Advanced or metastatic cancer.

-Use of Bevacizumab.

a cycle of CAPEOX consists of the following: capecitabine 1000 mg/m^2^ p.o. twice daily for 14 out of 21 days.

b A cycle of FOLFOX consists of the following: Day 1: i.v. oxaliplatin 85 mg/m^2^ in 120 min; i.v. leucovorin 200 mg/m^2^ in 2 h; 5-FU 400 mg/m^2^ bolus i.v. then 2400 mg/m^2^ perfusion i.v. over 46 h; The next chemotherapy cycle was repeated on the 15th day.

c A cycle of FOLFOXIRI consists of the following: Day 1: i.v. irinotecan 165 mg/m^2^ in 90 min; i.v. oxaliplatin 85 mg/m^2^ in 120 min; i.v. leucovorin 200 mg/m^2^ in 2 h; 5-FU 400 mg/m^2^ bolus i.v. then 2400 mg/m^2^ perfusion i.v. over 46 h; The next chemotherapy cycle was repeated on the 15th day.

### Predictors for recurrent VTE

In the competing-risk univariate analysis, 20 potential predictors of VTE recurrence were identified (**Table 1**). These variables were further evaluated in a multivariable analysis. Independent predictors of recurrence included age, history of VTE, index PE (with or without DVT), ECOG performance status ≥ 2, major surgery or trauma in the previous month, tumor stages II and III, hemoglobin < 100 g/L, and aPTT > 36.5 seconds (**Table 4**). The results of the univariate and multivariate models solely focusing on recurrence are also presented in **Table 4**. The proportions of thrombotic recurrences were stratified by different primary tumor sites, initial VTE events, and tumor stages, as shown in **Supplementary Tables S1–S3**. Predicted recurrence rates within these risk strata, accounting for death as a competing event, were estimated and visualized using cumulative incidence plots (**Supplementary Figures S2A–D, S3A–C, S4A–D**).

**Table 4:** Predictors for recurrent VTE at multivariate analysis.

Predictive variables for recurrent VTE	Multivariable model			Competing-risk multivariable model		
	Wald χ^2^	HR (95% CI)	*p*-value	Wald χ^2^	sHR (95% CI)	*p*-value
Age	8.871	1.005 (1.002-1.008)	0.003	12.012	1.005 (1.002-1.008)	0.001
BMI, > 28 kg/m^2^	0.335	1.133 (0.743-1.726)	0.563	0.756	1.192 (0.802-1.773)	0.385
History of VTE	63.833	5.050 (3.394-7.512)	< 0.001	53.463	4.288 (2.902-6.334)	< 0.001
Index PE (with or without DVT)	11.721	1.752 (1.271-2.415)	0.001	11.612	1.698 (1.252-2.303)	0.001
Isolated distal DVT	0.274	0.927 (0.699-1.230)	0.601	0.056	0.969 (0.747-1.257)	0.813
ECOG performance status ≥ 2	0.421	1.149 (0.756-1.745)	0.516	4.540	1.561 (1.036-2.350)	0.033
Major surgery or trauma in previous month	5.911	0.475 (0.261-0.866)	0.015	7.911	0.451 (0.259-0.786)	0.005
Tumor stage II	2.507	0.669 (0.407-1.100)	0.113	4.210	0.607 (0.377-0.978)	0.040
Tumor stage III	2.411	0.652 (0.381-1.118)	0.120	4.647	0.562 (0.333-0.949)	0.031
Metastatic cancer	0.116	0.900 (0.493-1.645)	0.733	1.028	0.742 (0.416-1.321)	0.311
Ongoing or recent chemotherapy	4.804	1.344 (1.032-1.751)	0.028	2.260	1.205 (0.945-1.538)	0.133
Hemoglobin < 100 g/L	2.178	1.241 (0.932-1.654)	0.140	5.224	1.363 (1.045-1.778)	0.022
CRP, mg/L	0.611	1.001 (0.998-1.004)	0.434	0.891	1.001 (0.999-1.004)	0.345
Creatinine clearance ≥ 90 mL/min	0.009	1.018 (0.697-1.487)	0.927	0.053	1.043 (0.730-1.490)	0.817
Creatinine clearance ≥ 60 and < 90 mL/min	0.494	0.883 (0.624-1.249)	0.482	0.034	0.970 (0.701-1.342)	0.854
PT > 12.5 sec	0.051	1.042 (0.726-1.497)	0.822	0.767	1.158 (0.834-1.607)	0.381
aPTT > 36.5 sec	6.722	2.047 (1.191-3.518)	0.010	7.474	2.034 (1.223-3.383)	0.006
INR > 1.2	1.649	1.347 (0.855-2.123)	0.199	0.581	1.175 (0.776-1.779)	0.446
Fibrin D-dimer, mg/L	0.986	0.979 (0.939-1.021)	0.321	0.990	0.981 (0.944-1.019)	0.320
Ottawa score ≥ 1	0.247	1.065 (0.831-1.364)	0.619	0.656	1.099 (0.874-1.382)	0.418

aPTT, activated partial thromboplastin time; BMI, body mass index; CI, confidence interval; CRP, C-reactive protein; DVT, deep venous thrombosis; ECOG, Eastern Cooperative Oncology Group; HR, hazard ratio; INR, international normalized ratio; PE, pulmonary embolism; PT, prothrombin time; sHR, sub-distribution hazard ratio; VTE, venous thromboembolism.

Death is considered as competing risk in sHR calculation.

The multivariate model employed a significance level of 0.05.

HR and sHR, along with their corresponding 95% CIs and *p*-values, were derived from a Cox proportional hazards multivariate model, using only the predictor as the model’s covariate.

Both continuous and categorical forms of the covariates were evaluated. If both were found to be significant, preference was given to the categorical forms for use in the multivariate models.

### Predictors for bleeding outcomes

In the competing-risk univariate Cox model, 17 potential predictors of MB were identified (**Table 2**), while 14 potential predictors of CRNMB were identified (**Table 3**). These variables were then evaluated in a multivariable analysis. Independent predictors for MB included age ≥ 75, history of MB, ECOG performance status ≥ 2, concomitant antiplatelet therapy, tumor stage III, metastatic cancer, and platelet counts < 100 K/μL (**Table 5**). Among these, three predictors (age ≥ 75, ECOG performance status ≥ 2, and platelet counts < 100 K/μL) were common risk factors for CRNMB, with hemoglobin < 100 g/L identified as an additional predictor for CRNMB (**Table 6**). The results of univariate and multivariate models considering only bleeding outcomes are shown in **Tables 2** and **3**. The proportions of bleeding events stratified by primary tumor site, initial VTE events, and tumor stages are detailed in **Supplementary Tables S1–S3**. Predicted rates of bleeding outcomes within these risk strata, accounting for death as a competing event, were estimated and visualized using cumulative incidence plots (**Supplementary Figures S2E–L, S3D–I, S4E–L**).

**Table 5:** Predictors for MB at multivariate analysis.

Predictive variables for MB	Multivariable model			Competing-risk multivariable model		
	Wald χ^2^	HR (95% CI)	*p*-value	Wald χ^2^	sHR (95% CI)	*p*-value
Age ≥ 75	0.052	1.090 (0.519-2.290)	0.820	4.018	1.637 (1.011-2.652)	0.045
History of major bleeding	10.903	8.208 (2.353-28.635)	0.001	9.923	5.320 (1.880-15.050)	0.002
Index PE (with or without DVT)	5.533	2.411 (1.158-5.019)	0.019	3.219	1.653 (0.955-2.862)	0.073
ECOG performance status ≥ 2	2.135	2.502 (0.731-8.562)	0.144	28.555	9.979 (4.292-23.203)	< 0.001
Major surgery or trauma in previous month	0.799	0.521 (0.125-2.178)	0.371	1.940	0.488 (0.178-1.340)	0.164
Concomitant antiplatelet therapy	15.444	3.942 (1.989-7.812)	< 0.001	12.823	2.592 (1.539-4.367)	< 0.001
Tumor stage III	9.299	0.137 (0.038-0.492)	0.002	25.034	0.122 (0.053-0.278)	< 0.001
Metastatic cancer	1.889	0.421 (0.122-1.446)	0.169	16.730	0.190 (0.086-0.421)	< 0.001
Primary tumor site, right colon	3.042	1.747 (0.933-3.272)	0.081	2.543	1.426 (0.922-2.206)	0.111
Bevacizumab use	0.008	1.045 (0.395-2.764)	0.929	1.715	1.471 (0.826-2.621)	0.190
Platelet counts < 100 K/μL	2.353	2.422 (0.782-7.503)	0.125	7.692	2.685 (1.336-5.397)	0.006
Hemoglobin < 100 g/L	0.841	1.397 (0.684-2.852)	0.359	3.446	1.561 (0.975-2.498)	0.063
CRP, mg/L	3.444	1.006 (1.000-1.012)	0.063	3.095	1.004 (1.000-1.008)	0.079
PT > 12.5 sec	0.001	0.988 (0.394-2.480)	0.980	0.731	1.288 (0.721-2.300)	0.393
aPTT > 36.5 sec	2.247	2.535 (0.751-8.555)	0.134	2.992	2.056 (0.909-4.653)	0.084
INR > 1.2	0.004	1.038 (0.339-3.185)	0.947	0.069	0.910 (0.451-1.837)	0.793
Fibrin D-dimer, mg/L	0.026	1.008 (0.914-1.112)	0.871	0.142	1.012 (0.951-1.077)	0.706

aPTT, activated partial thromboplastin time; CI, confidence interval; n; DVT, deep venous thrombosis; ECOG, Eastern Cooperative Oncology Group; HR, hazard ratio; INR, international normalized ratio; MB, major bleeding; PE, pulmonary embolism; PT, prothrombin time; sHR, sub-distribution hazard ratio; VTE, venous thromboembolism.

Death is considered as competing risk in sHR calculation.

The multivariate model employed a significance level of 0.05.

HR and sHR, along with their corresponding 95% CIs and p-values, were derived from a Cox proportional hazards multivariate model, using only the predictor as the model’s covariate.

Both continuous and categorical forms of the covariates were evaluated. If both were found to be significant, preference was given to the categorical forms for use in the multivariate models.

**Table 6:** Predictors for CRNMB at multivariate analysis.

Predictive variables for CRNMB	Multivariable model			Competing-risk multivariable model		
	Wald χ^2^	HR (95% CI)	*p*-value	Wald χ^2^	sHR (95% CI)	*p*-value
Age ≥ 75	0.185	1.111 (0.687-1.797)	0.667	3.934	1.465 (1.005-2.137)	0.047
BMI < 18.5 kg/m^2^	3.938	1.679 (1.006-2.800)	0.047	3.198	1.496 (0.962-2.325)	0.074
ECOG performance status ≥ 2	1.876	1.359 (0.876-2.110)	0.171	7.867	1.750 (1.184-2.586)	0.005
Metastatic cancer	0.442	0.865 (0.565-1.325)	0.506	0.030	1.031 (0.731-1.453)	0.864
WBC counts, K/μL	1.833	1.012 (0.995-1.030)	0.176	2.243	1.011 (0.997-1.026)	0.134
Platelet counts < 100 K/μL	4.170	2.187 (1.032-4.633)	0.041	4.761	2.057 (1.076-3.932)	0.029
Hemoglobin < 100 g/L	8.819	1.886 (1.241-2.868)	0.003	12.175	1.870 (1.316-2.657)	< 0.001
CRP, mg/L	0.070	1.001 (0.996-1.005)	0.792	0.154	1.001 (0.997-1.004)	0.695
PT, sec	2.000	1.657 (0.823-3.335)	0.157	0.117	1.111 (0.606-2.038)	0.732
aPTT, sec	0.632	1.012 (0.982-1.044)	0.426	2.731	1.027 (0.995-1.061)	0.098
INR	1.836	0.005 (0.000-10.449)	0.175	0.069	0.410 (0.001-311.165)	0.792
INR > 1.2	0.968	0.716 (0.368-1.393)	0.325	0.569	0.816 (0.481-1.384)	0.451
Fibrinogen < 2.00 g/L	0.091	1.186 (0.391-3.604)	0.763	0.018	1.069 (0.407-2.806)	0.892
Fibrin D-dimer, mg/L	1.287	1.037 (0.974-1.103)	0.257	2.176	1.040 (0.987-1.095)	0.140

aPTT, activated partial thromboplastin time; BMI, body mass index; CI, confidence interval; CRNMB, clinical relevant non major bleeding; CRP, C-reactive protein; ECOG, Eastern Cooperative Oncology Group; HR, hazard ratio; INR, international normalized ratio; PT, prothrombin time; RBC, red blood cell; sHR, sub-distribution hazard ratio; WBC, white blood cell.

Death is considered as competing risk in sHR calculation.

The multivariate model employed a significance level of 0.05.

HR and sHR, along with their corresponding 95% CIs and p-values, were derived from a Cox proportional hazards multivariate model, using only the predictor as the model’s covariate.

Both continuous and categorical forms of the covariates were evaluated. If both were found to be significant, preference was given to the categorical forms for use in the multivariate models.

### Anticoagulant outcomes and mortality

**Figure 2:**
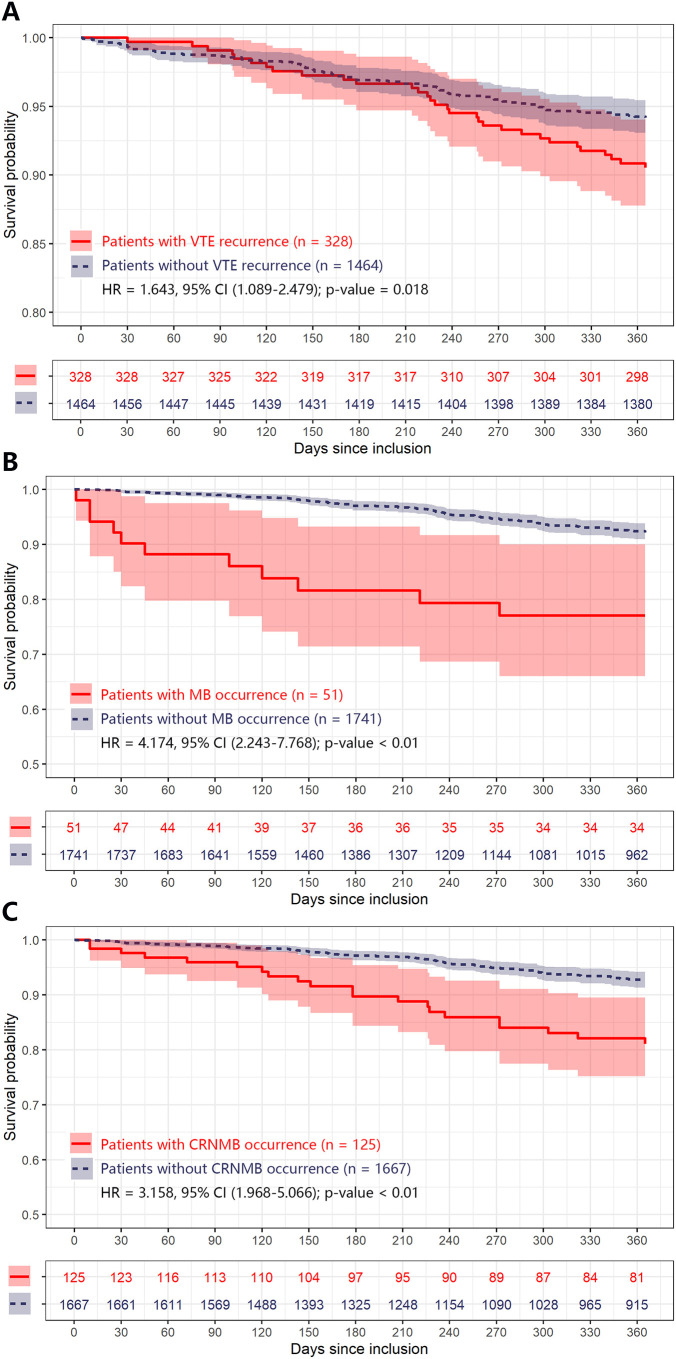
Kaplan-Meier plot of estimated one-year mortality according to different adverse anticoagulant outcomes in CRC patients. Any differences in the incidence were evaluated with a log-rank test. Plot **(A–C)** were grouped by patients with different adverse anticoagulant outcomes (including VTE recurrence, MB, and CRNMB) during one-year follow, respectively. CI, confidence interval; CRC, colorectal cancer; CRNMB, clinical relevant non major bleeding; HR, hazard ratio; MB, major bleeding; VTE, venous thromboembolism.

The influence of VTE recurrence and bleeding on mortality is shown in **Table 7**. In cancer patients undergoing anticoagulant therapy, recurrent VTE was associated with an over 60% increase in one-year mortality rates, with a HR of 1.643 (95% CI 1.089-2.479). Likewise, bleeding events significantly increased mortality, with HRs of 4.174 (95% CI 2.243-7.768) for MB and 3.158 (95% CI 1.968-5.066) for CRNMB. The results from the Kaplan-Meier survival curves are shown in **Figure 2**.

**Table 7:** Impact of recurrence thrombosis or bleeding on mortality outcomes.

Outcomes during anticoagulant therapy	Hazard ratio (95% CI)	*p*-value
Recurrent venous thrombosis	1.643 (1.089-2.479)	0.018
Major bleeding	4.174 (2.243-7.768)	< 0.01
Clinically relevant non major bleeding	3.158 (1.968-5.066)	< 0.01

CI, confidence interval.

## Discussion

This analysis of clinical and laboratory data in CRC patients with cancer-associated VTE, who received standardized anticoagulant treatment in a real-world setting, identified several independent predictors through competing-risk multivariate analysis. In this study, the incidence of VTE recurrence and total bleeding (including MB and CRNMB) was approximately 18% and 10%, respectively. A recent multicenter cohort study of cancer patients reported a cumulative VTE recurrence incidence of 11.4% at six months during anticoagulant treatment (18). Another prospective cohort study observed a high bleeding rate of 11.4% in cancer patients receiving anticoagulants (7). However, in rigorous clinical trials of anticoagulants, VTE recurrence rates ranged from 3.5% to 9.6%, and MB rates varied between 0.7% and 5.4% (13, 19–22). The higher recurrence and bleeding rates observed in this study may be attributed to the real-world nature of the cohort, including spontaneous discontinuation of anticoagulant therapy due to bleeding concerns and the lack of tolerability in CRC patients.

Our results showed that each of the outcomes (VTE recurrence, MB, and CRNMB) negatively impacts patient survival (**Figure 2**, **Table 7**). Such effects on VTE patients with various cancer types have been evaluated by other researchers. Lecumberri et al. used the RIETE registry to investigate mortality rates among VTE patients during anticoagulant treatment (23). The overall mortality rate for recurrent VTE patients was 12.1%, with recurrent PE patients having a higher rate (18.5%) compared to recurrent DVT patients (6.3%). The overall mortality rate for MB patients was 19.7%, rising to 24.4% in the subgroup of cancer patients, which is in line with our results (approximately 22%). However, a previous systematic review reported identical mortality rates of 11.3% for both recurrent VTE and MB patients within the first three months of anticoagulant therapy (24). More recently, a prospective cohort study indicated that episodes of VTE recurrence, MB, and CRNMB increased the overall mortality rate in cancer patients by more than 50%, 80%, and 30%, respectively (7). This finding is also consistent with our data. Given the negative impact of these adverse events on prognosis, it is crucial to identify cancer patients at the highest risk of such outcomes, especially those with cancer types like CRC that have high rates of VTE and bleeding.

A history of VTE and bleeding are well-established predictors of recurrence and bleeding in cancer-associated VTE patients (8–10). In our multivariate model, index PE was identified as a significant risk factor for VTE recurrence. However, similar tendencies were not observed in patients with index proximal DVT or isolated distal DVT. Previous studies have shown that residual pulmonary artery obstructions after anticoagulant treatment for PE or DVT can predict recurrent VTE (25–27). In the Vienna prediction model for non-cancer patients, index proximal DVT and index PE were identified as risk factors for VTE recurrence, with HRs of 2.08 (95% CI 1.16-3.74) and 2.60 (95% CI 1.49-4.53), respectively, compared to index distal DVT (28). A recent study including both cancer and non-cancer patients reported a similar HR direction for index PE (1.02, 95% CI 0.89-1.18), though it was not statistically significant (29).

Surgery is recognized as a significant but temporary risk factor for VTE and is typically associated with a low likelihood of recurrence (30). In our study, it was found that surgery was associated with a lack of recurrence even in the presence of active cancer. This finding aligns with a multicenter cohort study that found cancer patients who underwent surgery within the previous two months had a reduced risk of recurrence, with a HR of 0.60 (95% CI 0.40-0.92) (31). Another cohort study investigated surgery at the time of incident VTE as a predictor of recurrence but did not find statistically significant results (32). On the other hand, the association between an ECOG performance status of 1 or higher and an increased rate of recurrent VTE is clinically plausible, reflecting more advanced cancer disease.

In clinical practice, the potential benefits of anticoagulant therapy are typically compromised by the increased bleeding risk in cancer patients, particularly those with CRC. This bleeding risk is further exacerbated by malignancy-related conditions and treatments (33–35). To complicate matters, some predictors for recurrence are also correlated with an increased risk of bleeding (10, 36). Our analysis revealed limited overlap between the risks of VTE recurrence and bleeding in CRC patients. Notably, age and ECOG status were the only two common risk factors for both VTE recurrence and bleeding. Specifically, age over 75 years was a significant risk factor for both MB and CRNMB, while age was a continuous variable risk factor for VTE recurrence. Hemoglobin levels less than 100 g/L emerged as a common risk factor for VTE recurrence and CRNMB in the multivariate analysis. A similar trend was observed for MB, although this did not reach statistical significance (sHR = 1.561, 95% CI 0.975-2.498).

It is generally believed that the incidence of VTE is variable between different cancer types (8–10). Due to the apparent differences in blood supply and environmental carcinogens among different primary tumor locations in CRC (37, 38), it is presumed that VTE recurrence and bleeding risks might also differ by anatomical subsites. However, our multivariate analysis did not reveal any statistically significant correlations. Only patients with primary tumors in the right colon showed a higher risk of MB in the univariate analysis (sHR = 1.87, 95% CI 1.23-2.85). Similarly, we found no evidence linking portal venous thrombosis (PVT) with anticoagulant outcomes, despite previous studies indicating that patients with PVT are at risk for bleeding, and thrombosis recurrence in both splanchnic veins and deep veins of the lower extremities, as well as pulmonary artery (39, 40).

Risk factors for VTE recurrence and bleeding have been used to develop various risk scores. In our cohort of CRC patients, we evaluated the Ottawa score (41), which was specifically designed to identify cancer patients at risk for VTE recurrence (**Supplementary Table S4**). Although the 1067 low-risk patients (score < 1) exhibited a numerically lower cumulative incidence of recurrent VTE compared to high-risk patients (16.7% vs. 20.5%, respectively), the C-index value was still relatively low (0.60). This finding aligns with a previous prospective multicenter study involving patients with various cancer types, which also reported the insufficient accuracy of the Ottawa score in predicting recurrent VTE (42). Previous studies have demonstrated that a diagnosis of gastrointestinal cancer is an independent risk factor for both MB and CRNMB in cancer patients (8, 10, 11). For cancer-associated VTE patients, due to the absence of prospectively validated bleeding scores, we selected four well-recognized bleeding risk factors for analysis. However, no meaningful correlations were found (**Tables 2**, **3**). Further investigation into bleeding outcome prediction in cancer-associated VTE patients is warranted.

This study has several limitations. First, the retrospective design does not allow for the inclusion over treatment-related parameters typical of a randomized controlled trial. While the univariate analysis identified the thrombogenic effects of concurrent anticancer treatments, the diverse range of anticancer agents and their varying administration among patients prevented the correlating of recurrence and bleeding risk with any specific agent. Importantly, our study focused specifically on a large real-world cohort of CRC patients with VTE, who are underrepresented in prior research. Furthermore, we found that the rates of VTE recurrence and bleeding in CRC patients under routine clinical care were higher than those reported in major trials, highlighting important real-world challenges and the clinical significance of our findings. Another limitation of this study is that only rivaroxaban and LMWH were analyzed, as other DOACs were not accessible at our center during the study period. Third, the use of DOACs is often discouraged in patients with gastrointestinal or genitourinary cancers, which may introduce bias in future studies that compare outcomes in cancer-associated VTE patients treated with these anticoagulants.

## Conclusions

This study identified risk factors for VTE recurrence and bleeding in CRC patients undergoing anticoagulant therapy. Our analysis demonstrated the negative impact of these outcomes on survival, highlighting the need for improved stratification methods for CRC patients. Furthermore, the limited overlap between risk factors for recurrence and bleeding observed in our analysis may aid in the development of more effective risk prediction models in CRC patients, thereby enhancing the overall risk-benefit assessment of anticoagulant treatment in this population.

## Data Availability

The raw data supporting the conclusions of this article will be made available by the authors, without undue reservation.
