# Supplementary material for: Risk factors for recurrence and bleeding in colorectal cancer patients with cancer-associated venous thrombembolism
Source: Front Oncol. 2025 Aug 27;15:1648003. doi: 10.3389/fonc.2025.1648003 (PMC12420301; doi:10.3389/fonc.2025.1648003)
Supplement: Supplementary file 1 [file DataSheet1.docx]

**
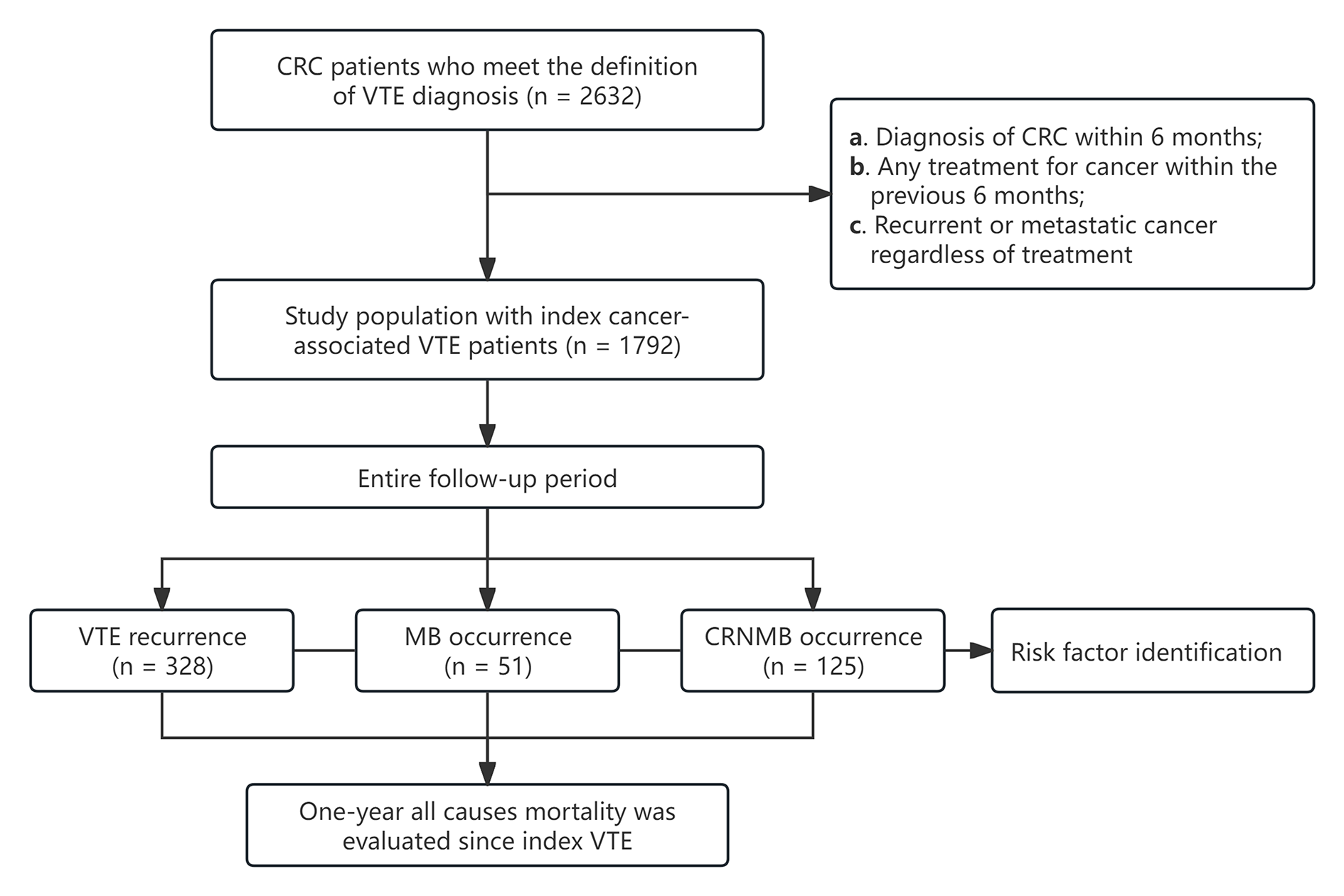
**

**Supplementary Figure S1** Flowchart illustrating participant selection into the datasets.

**
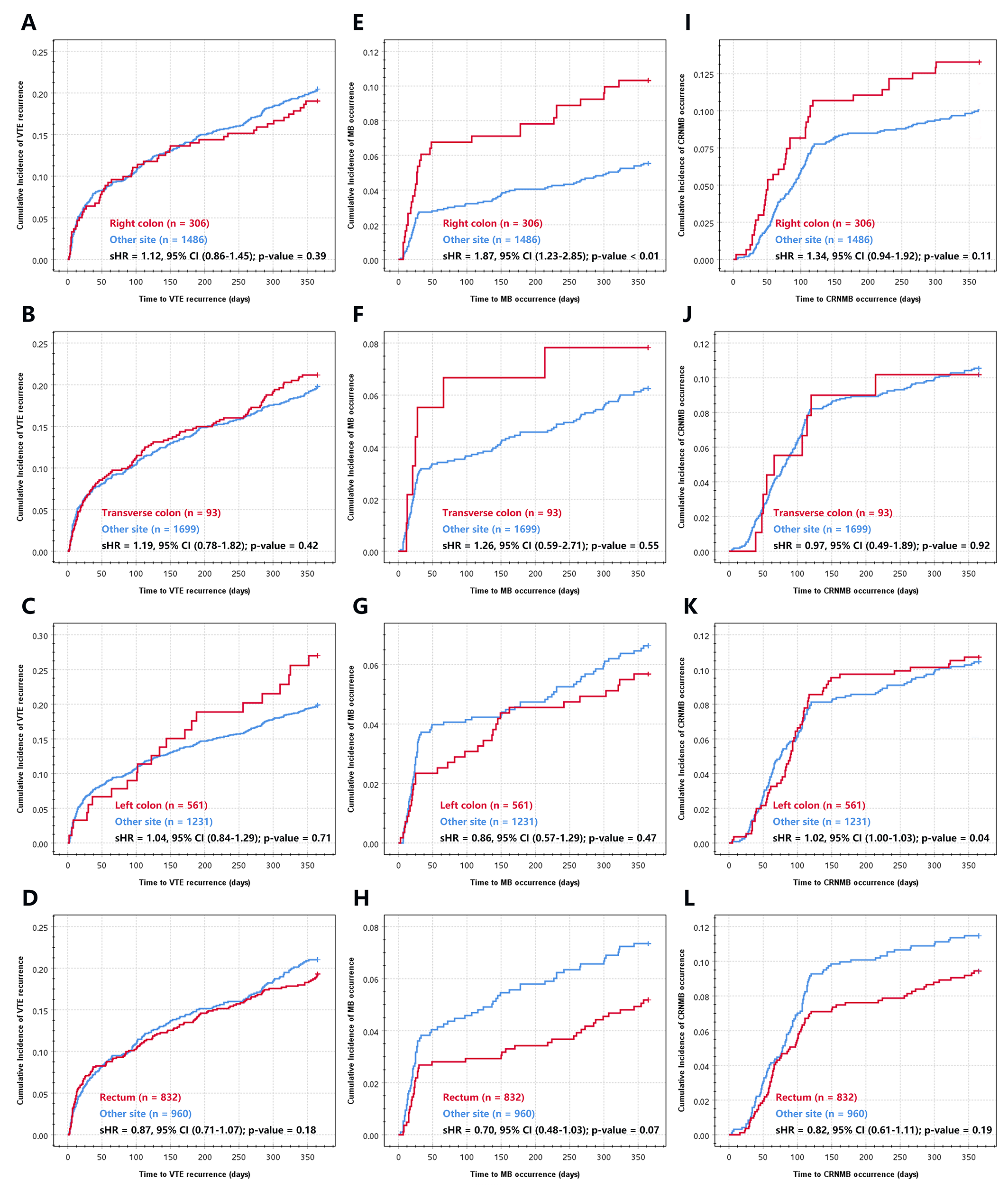
**

**Supplementary Figure S2** Cumulative incidence curves of VTE recurrence (plot A, B, C, and D), MB (plot E, F, G, and H), and CRNMB (plot I, J, K, and L) in CRC patients with different primary tumor sites (including right colon, transverse colon, left colon, and rectum). The sHR value of each group was estimated by Fine and Gray method considering all cause death as competing event. Any differences in the incidence were evaluated with a log-rank test. CI, confidence interval; CRC, colorectal cancer; CRNMB, clinical relevant non major bleeding; MB, major bleeding; sHR, sub-distribution hazard ratio; VTE, venous thromboembolism.


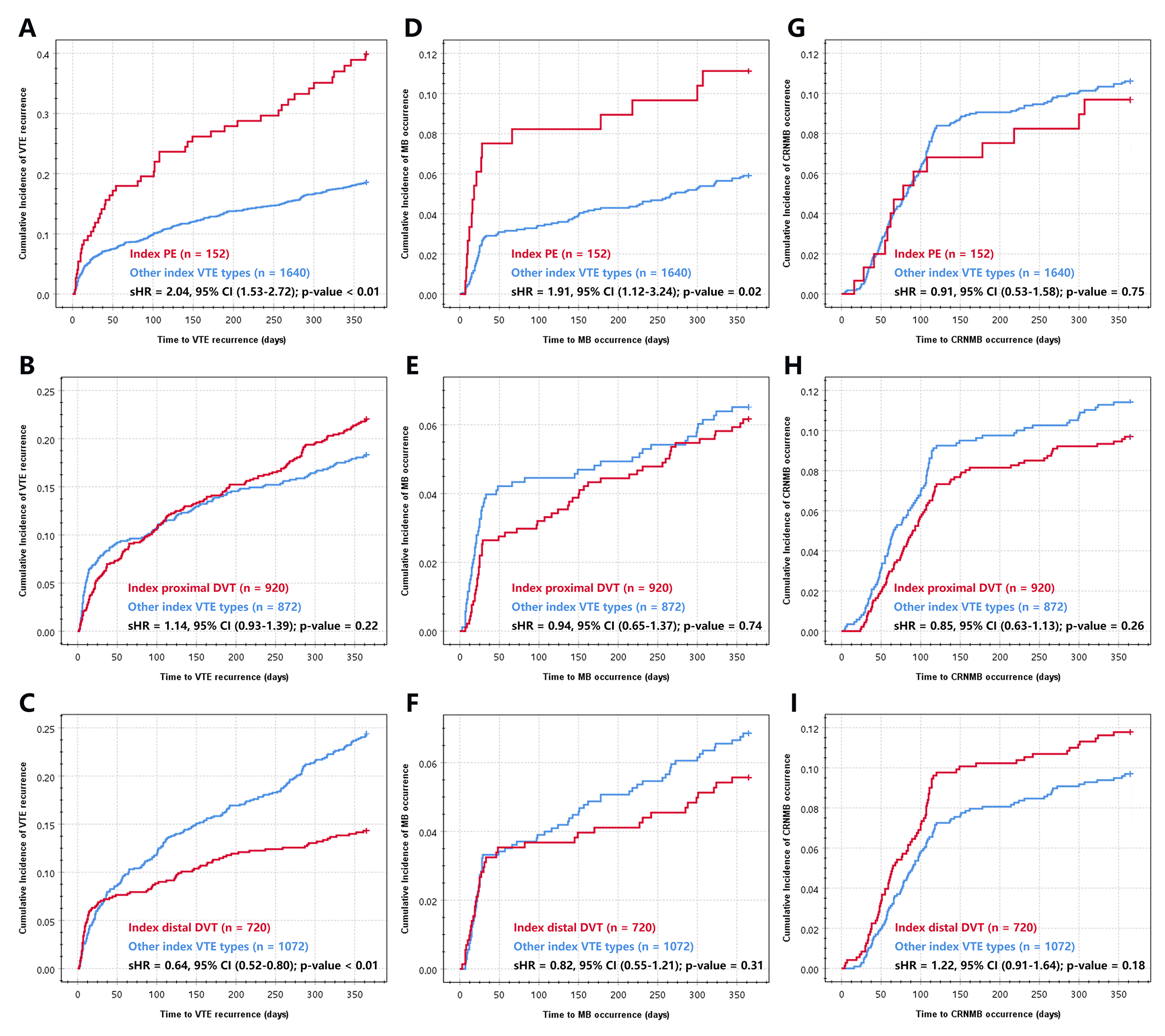


**Supplementary Figure S3** Cumulative incidence curves of VTE recurrence (plot A, B, and C), MB (plot D, E, and F), and CRNMB (plot G, H, and I) in CRC patients with different types of index VTE (including index PE, index proximal DVT, and index distal DVT). The sHR value of each group was estimated by Fine and Gray method considering all cause death as competing event. Any differences in the incidence were evaluated with a log-rank test. CI, confidence interval; CRC, colorectal cancer; CRNMB, clinical relevant non major bleeding; DVT, deep venous thrombosis; MB, major bleeding; sHR, sub-distribution hazard ratio; VTE, venous thromboembolism; PE, pulmonary embolism.


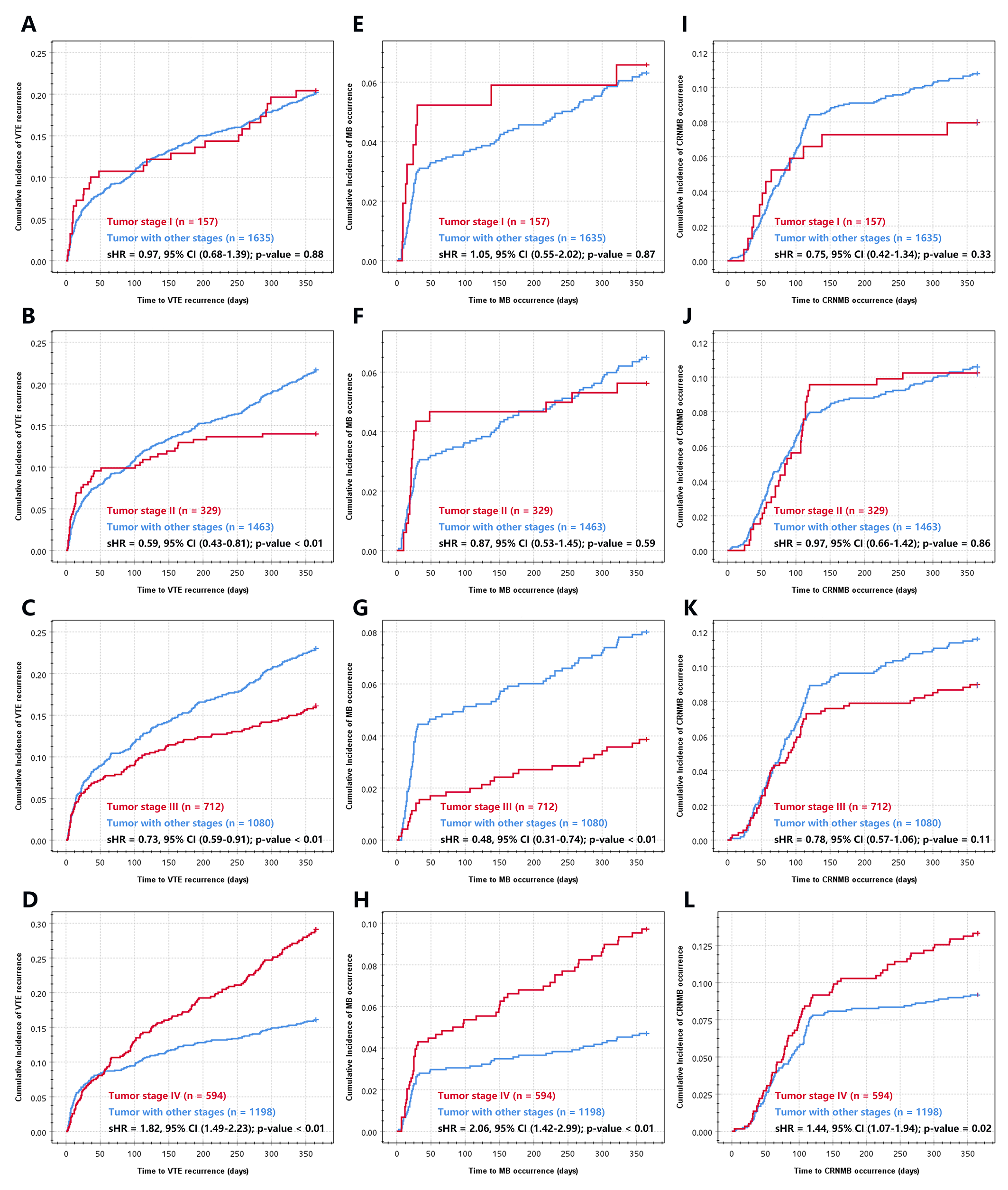


**Supplementary Figure S4** Cumulative incidence curves of VTE recurrence (plot A, B, C, and D), MB (plot E, F, G, and H), and CRNMB (plot I, J, K, and L) in CRC patients with different primary tumor sites (including right colon, transverse colon, left colon, and rectum). The sHR value of each group was estimated by Fine and Gray method considering all cause death as competing event. Any differences in the incidence were evaluated with a log-rank test. CI, confidence interval; CRC, colorectal cancer; CRNMB, clinical relevant non major bleeding; MB, major bleeding; sHR, sub-distribution hazard ratio; VTE, venous thromboembolism.

**Supplementary Table S1** Actual cumulative incidence of recurrent VTE, MB, and CRNMB at one year according to primary tumor site

| **Primary site**  **of CRC** | **Number**  **of patients** | **Recurrent VTE, n% (95% CI)** | | **MB, n% (95% CI)** | | **CRNMB, n% (95% CI)** | |
| --- | --- | --- | --- | --- | --- | --- | --- |
| Right colon | 306 | 53, 17.3% (12.9-21.7) | | 15, 4.9% (2.6-7.4) | | 25, 8.2% (5.2-11.4) | |
|  |  | Type of recurrent VTE | | Location of bleeding source | | Location of bleeding source | |
|  |  | PE with or without DVT | 6, 2.0% (0.6-3.5) | Gastrointestinal^a^ | 8, 2.6% (1.0-4.4) | Gastrointestinal^c^ | 11, 3.6% (1.7-5.7) |
|  |  | Isolated distal DVT | 22, 7.2% (4.4-10.1) | Urologic^a^ | 2, 0.7% (0.1-1.8) | Urologic^c^ | 5, 1.6% (0.3-3.2) |
|  |  | Proximal DVT with or without distal DVT | 25, 8.2% (5.2-11.4) | Pulmonary^a^ | 0 | Gynecological^c^ | 4, 1.3% (0.3-2.6) |
|  |  |  |  | Critical sites^b^ | 5, 1.6% (0.3-3.1) | Other sites^d^ | 6, 2.0% (0.6-3.6) |
| Transverse colon | 93 | 22, 23.7% (15.4-32.6) | | 5, 5.4% (1.2-10.5) | | 7, 7.5% (2.4-13.5) | |
|  |  | Type of recurrent VTE | | Location of bleeding source | | Location of bleeding source | |
|  |  | PE with or without DVT | 3, 3.2% (1.0-7.2) | Gastrointestinal^a^ | 3, 3.2% (0.1-7.4) | Gastrointestinal^c^ | 3, 3.2% (0.1-7.7) |
|  |  | Isolated distal DVT | 5, 5.4% (1.1-10.5) | Urologic^a^ | 0 | Urologic^c^ | 0 |
|  |  | Proximal DVT with or without distal DVT | 14, 15.1% (7.8-22.6) | Pulmonary^a^ | 0 | Gynecological^c^ | 1, 1.1% (0.1-3.6) |
|  |  |  |  | Critical sites^b^ | 2, 2.2% (0.1-5.3) | Other sites^d^ | 3, 3.2% (0.1-7.1) |
| Left colon | 561 | 107, 19.1% (15.9-22.4) | | 12, 2.1% (1.0-3.3) | | 41, 7.3% (5.4-9.5) | |
|  |  | Type of recurrent VTE | | Location of bleeding source | | Location of bleeding source | |
|  |  | PE with or without DVT | 13, 2.3% (1.2-3.6) | Gastrointestinal^a^ | 6, 1.1% (0.3-1.9) | Gastrointestinal^c^ | 20, 3.6% (2.1-5.2) |
|  |  | Isolated distal DVT | 29, 5.2% (3.3-7.0) | Urologic^a^ | 2, 0.4% (0.1-0.9) | Urologic^c^ | 6, 1.1% (0.3-2.1) |
|  |  | Proximal DVT with or without distal DVT | 65, 11.6% (8.7-14.2) | Pulmonary^a^ | 2, 0.4% (0.1-0.9) | Gynecological^c^ | 7, 1.2% (0.4-2.2) |
|  |  |  |  | Critical sites^b^ | 2, 0.4% (0.1-0.9) | Other sites^d^ | 8, 1.4% (0.5-2.5) |
| Rectum | 832 | 146, 17.5% (15.0-20.3) | | 19, 2.3% (1.3-3.4) | | 52, 6.3% (4.7-8.0) | |
|  |  | Type of recurrent VTE | | Location of bleeding source | | Location of bleeding source | |
|  |  | PE with or without DVT | 9, 1.1% (0.4-1.8) | Gastrointestinal^a^ | 10, 1.2% (0.6-2.0) | Gastrointestinal^c^ | 24, 2.9% (1.8-4.1) |
|  |  | Isolated distal DVT | 53, 6.4% (4.9-8.1) | Urologic^a^ | 3, 0.4% (0.1-0.8) | Urologic^c^ | 11, 1.3% (0.6-2.1) |
|  |  | Proximal DVT with or without distal DVT | 84, 10.1% (8.2-12.1) | Pulmonary^a^ | 2, 0.2% (0.1-0.6) | Gynecological^c^ | 5, 0.6% (0.1-1.2) |
|  |  |  |  | Critical sites^b^ | 4, 0.5% (0.1-1.0) | Other sites^d^ | 16, 1.9% (1.1-2.9) |

CI, confidence interval; CRC, colorectal cancer; CRNMB, clinical relevant non major bleeding; DVT, deep venous thrombosis; MB, major bleeding; VTE, venous thromboembolism; PE, pulmonary embolism.

^a^Acute clinically overt bleeding associated with a decrease in the hemoglobin level of at least 2 g per deciliter and/or a transfusion of 2 or more units of red cells.

^b^Bleeding occurring at critical sites (intracranial, intraspinal, intraocular, pericardial, intraarticular, intramuscular with compartment syndrome, or retroperitoneal).

^c^Bleeding not meeting the criteria for MB but associated with medical intervention, unscheduled contact with a member of the health care team, or temporary cessation of the treatment.

^d^Bleeding occurring at other sites (ear, nose, throat, oral, cutaneous).

**Supplementary Table S2** Actual cumulative incidence of recurrent VTE, MB, and CRNMB at one year according to different index VTE events

| **Type Index VTE** | **Number**  **of patients** | **Recurrent VTE, n% (95% CI)** | | **MB, n% (95% CI)** | | **CRNMB, n% (95% CI)** | |
| --- | --- | --- | --- | --- | --- | --- | --- |
| PE with or without DVT | 152 | 50, 32.9% (26.0-40.5) | | 10, 6.6% (3.1-10.8) | | 8, 5.3% (2.0-8.8) | |
|  |  | Type of recurrent VTE | | Location of bleeding source | | Location of bleeding source | |
|  |  | PE with or without DVT | 8, 5.3% (1.9-9.3) | Gastrointestinal^a^ | 6, 3.9% (1.4-7.2) | Gastrointestinal^c^ | 2, 1.3% (0.1-3.4) |
|  |  | Isolated distal DVT | 20, 13.2% (8.2-18.8) | Urologic^a^ | 2, 1.3% (0.1-3.5) | Urologic^c^ | 1, 0.7% (0.1-2.1) |
|  |  | Proximal DVT with or without distal DVT | 22, 14.5% (8.8-20.4) | Pulmonary^a^ | 1, 0.7% (0.1-2.2) | Gynecological^c^ | 2, 1.3% (0.1-3.4) |
|  |  |  |  | Critical sites^b^ | 1, 0.7% (0.1-2.2) | Other sites^d^ | 3, 2.0% (0.1-4.5) |
| Isolated  distal DVT | 720 | 96, 13.3% (11.0-15.8) | | 19, 2.6% (1.5-3.9) | | 60, 8.3% (6.2-10.4) | |
|  |  | Type of recurrent VTE | | Location of bleeding source | | Location of bleeding source | |
|  |  | PE with or without DVT | 7, 1.0% (0.3-1.8) | Gastrointestinal^a^ | 8, 1.1% (0.4-2.0) | Gastrointestinal^c^ | 32, 4.4% (2.8-6.0) |
|  |  | Isolated distal DVT | 57, 7.9% (5.9-9.9) | Urologic^a^ | 4, 0.6% (0.1-1.2) | Urologic^c^ | 12, 1.7% (0.8-2.6) |
|  |  | Proximal DVT with or without distal DVT | 32, 4.4% (2.9-6.1) | Pulmonary^a^ | 0 | Gynecological^c^ | 5, 0.7% (0.1-1.4) |
|  |  |  |  | Critical sites^b^ | 7, 1.0% (0.3-1.8) | Other sites^d^ | 15, 2.1% (1.1-3.2) |
| Proximal DVT with or without distal DVT | 920 | 182, 19.8% (17.0-22.4) | | 22, 2.4% (1.4-3.5) | | 57, 6.2% (4.7-7.9) | |
|  |  | Type of recurrent VTE | | Location of bleeding source | | Location of bleeding source | |
|  |  | PE with or without DVT | 16, 1.7% (1.0-2.8) | Gastrointestinal^a^ | 13, 1.4% (0.7-2.2) | Gastrointestinal^c^ | 24, 2.6% (1.6-3.7) |
|  |  | Isolated distal DVT | 32, 3.5% (2.4-4.7) | Urologic^a^ | 1, 0.1% (0.1-0.3) | Urologic^c^ | 9, 1.0% (0.4-1.6) |
|  |  | Proximal DVT with or without distal DVT | 134, 14.6% (12.2-16.8) | Pulmonary^a^ | 3, 0.3% (0.1-0.7) | Gynecological^c^ | 10, 1.1% (0.4-1.9) |
|  |  |  |  | Critical sites^b^ | 5, 0.5% (0.1-1.1) | Other sites^d^ | 15, 1.6% (0.8-2.5) |

CI, confidence interval; CRC, colorectal cancer; CRNMB, clinical relevant non major bleeding; DVT, deep venous thrombosis; MB, major bleeding; VTE, venous thromboembolism; PE, pulmonary embolism.

^a^Acute clinically overt bleeding associated with a decrease in the hemoglobin level of at least 2 g per deciliter and/or a transfusion of 2 or more units of red cells.

^b^Bleeding occurring at critical sites (intracranial, intraspinal, intraocular, pericardial, intraarticular, intramuscular with compartment syndrome, or retroperitoneal).

^c^Bleeding not meeting the criteria for MB but associated with medical intervention, unscheduled contact with a member of the health care team, or temporary cessation of the treatment.

^d^Bleeding occurring at other sites (ear, nose, throat, oral, cutaneous).

**Supplementary Table S3** Actual cumulative incidence of recurrent VTE, MB, and CRNMB at one year according to different tumor stages

| **TNM stage** | **Number**  **of patients** | **Recurrent VTE, n% (95% CI)** | | **MB, n% (95% CI)** | | **CRNMB, n% (95% CI)** | |
| --- | --- | --- | --- | --- | --- | --- | --- |
| Stage I | 157 | 29, 18.5% (12.7-24.8) | | 6, 3.8% (1.3-7.0) | | 7, 4.5% (1.3-8.3) | |
|  |  | Type of recurrent VTE | | Location of bleeding source | | Location of bleeding source | |
|  |  | PE with or without DVT | 2, 1.3% (0.1-3.2) | Gastrointestinal^a^ | 3, 1.9% (0.1-4.2) | Gastrointestinal^c^ | 4, 2.5% (0.6-5.1) |
|  |  | Isolated distal DVT | 15, 9.6% (5.1-14.0) | Urologic^a^ | 1, 0.6% (0.1-2.1) | Urologic^c^ | 2, 1.3% (0.1-3.2) |
|  |  | Proximal DVT with or without distal DVT | 12, 7.6% (3.8-11.5) | Pulmonary^a^ | 1, 0.6% (0.1-2.2) | Gynecological^c^ | 2, 1.3% (0.1-3.2) |
|  |  |  |  | Critical sites^b^ | 1, 0.6% (0.1-2.1) | Other sites^d^ | 0 |
| Stage II | 329 | 43, 13.1% (9.4-17.0) | | 14, 4.3% (2.1-6.4) | | 29, 8.8% (5.8-11.9) | |
|  |  | Type of recurrent VTE | | Location of bleeding source | | Location of bleeding source | |
|  |  | PE with or without DVT | 6, 1.8% (0.6-3.3) | Gastrointestinal^a^ | 8, 2.4% (0.9-4.1) | Gastrointestinal^c^ | 17, 5.2% (3.0-7.6) |
|  |  | Isolated distal DVT | 18, 5.5% (3.0-7.9) | Urologic^a^ | 1, 0.3% (0.1-1.0) | Urologic^c^ | 4, 1.2% (0.3-2.7) |
|  |  | Proximal DVT with or without distal DVT | 19, 5.8% (3.3-8.5) | Pulmonary^a^ | 1, 0.3% (0.1-1.0) | Gynecological^c^ | 1, 0.3% (0.1-0.9) |
|  |  |  |  | Critical sites^b^ | 4, 1.2% (0.3-2.6) | Other sites^d^ | 8, 2.4% (0.9-4.3) |
| Stage III | 712 | 106, 14.9% (12.4-17.4) | | 7, 1.0% (0.3-1.7) | | 45, 6.3% (4.5-8.0) | |
|  |  | Type of recurrent VTE | | Location of bleeding source | | Location of bleeding source | |
|  |  | PE with or without DVT | 7, 1.0% (0.3-1.8) | Gastrointestinal^a^ | 1, 0.1% (0.1-0.4) | Gastrointestinal^c^ | 18, 2.5% (1.5-3.8) |
|  |  | Isolated distal DVT | 44, 6.2% (4.5-8.0) | Urologic^a^ | 3, 0.4% (0.1-1.0) | Urologic^c^ | 9, 1.3% (0.6-2.1) |
|  |  | Proximal DVT with or without distal DVT | 55, 7.7% (5.8-9.8) | Pulmonary^a^ | 1, 0.1% (0.1-0.4) | Gynecological^c^ | 5, 0.7% (0.1-1.4) |
|  |  |  |  | Critical sites^b^ | 2, 0.3% (0.1-0.7) | Other sites^d^ | 16, 2.2% (1.1-3.5) |
| Stage IV | 594 | 150, 25.3% (21.9-29.0) | | 24, 4.0% (2.5-5.6) | | 44, 7.4% (5.4-9.4) | |
|  |  | Type of recurrent VTE | | Location of bleeding source | | Location of bleeding source | |
|  |  | PE with or without DVT | 16, 2.7% (1.3-4.0) | Gastrointestinal^a^ | 15, 2.5%(1.3-3.8) | Gastrointestinal^c^ | 19, 3.2% (1.9-4.7) |
|  |  | Isolated distal DVT | 32, 5.4% (3.7-7.2) | Urologic^a^ | 2, 0.3% (0.1-0.8) | Urologic^c^ | 7, 1.2% (0.3-2.2) |
|  |  | Proximal DVT with or without distal DVT | 102, 17.2% (14.1-20.2) | Pulmonary^a^ | 1, 0.2% (0.1-0.5) | Gynecological^c^ | 9, 1.5% (0.7-2.5) |
|  |  |  |  | Critical sites^b^ | 6, 1.0% (0.3-1.9) | Other sites^d^ | 9, 1.5% (0.7-2.5) |

CI, confidence interval; CRC, colorectal cancer; CRNMB, clinical relevant non major bleeding; DVT, deep venous thrombosis; MB, major bleeding; VTE, venous thromboembolism; PE, pulmonary embolism.

^a^Acute clinically overt bleeding associated with a decrease in the hemoglobin level of at least 2 g per deciliter and/or a transfusion of 2 or more units of red cells.

^b^Bleeding occurring at critical sites (intracranial, intraspinal, intraocular, pericardial, intraarticular, intramuscular with compartment syndrome, or retroperitoneal).

^c^Bleeding not meeting the criteria for MB but associated with medical intervention, unscheduled contact with a member of the health care team, or temporary cessation of the treatment.

^d^Bleeding occurring at other sites (ear, nose, throat, oral, cutaneous).

**Supplementary Table S4** The Ottawa score for recurrent VTE risk in cancer-associated thrombosis

| **Variable** |  | **Point** |
| --- | --- | --- |
| Gender | Female | 1 |
| Primary tumor site | Lung | 1 |
|  | Breast | -1 |
| TNM stage | Stage I | -2 |
| Previous VTE | Yes | 1 |
| Clinical probability |  | Low (< 1) |
|  |  | High (≥ 1) |

TNM, tumor nodes metastasis staging system (solid tumor only); VTE, venous thromboembolism.
